# Transcriptomic profiling after B cell depletion reveals central and peripheral immune cell changes in multiple sclerosis

**DOI:** 10.1172/JCI182790

**Published:** 2025-03-11

**Authors:** Jessica Wei, Jeonghyeon Moon, Yoshiaki Yasumizu, Le Zhang, Khadir Radassi, Nicholas Buitrago-Pocasangre, M. Elizabeth Deerhake, Nicolas Strauli, Chun-Wei Chen, Ann Herman, Rosetta Pedotti, Catarina Raposo, Isaiah Yim, Jenna Pappalardo, Erin E. Longbrake, Tomokazu S. Sumida, Pierre-Paul Axisa, David A. Hafler

**Affiliations:** 1 Department of Neurology and; 2 Department of Immunobiology, Yale School of Medicine, New Haven, Connecticut, USA.; 3 Department of Experimental Immunology, Immunology Frontier Research Center, Osaka University, Suita, Osaka, Japan.; 4 Genentech, San Francisco, California, USA.; 5 Roche, Basel, Switzerland.; 6 Department of Biomedical Engineering, Yale University, New Haven, Connecticut, USA.; 7 Centre de recherches en cancérologie de Toulouse (CRCT), INSERM, Toulouse, France.; 8 Broad Institute, Boston, Massachusetts, USA.

**Keywords:** Autoimmunity, Immunology, Multiple sclerosis

## Abstract

Multiple sclerosis (MS) is a complex, genetically mediated autoimmune disease of the CNS, in which anti-CD20–mediated B cell depletion is remarkably effective in the treatment of early disease. Although previous studies investigated the effect of B cell depletion on select immune cell subsets using flow cytometry–based methods, the therapeutic effect on the patient’s immune landscape is unknown. In this study, we explored how B cell–depleting therapies modulate the immune landscape using single-cell RNA-Seq. We demonstrate that B cell depletion led to cell-type–specific changes in the abundance and function of cerebrospinal fluid (CSF) macrophages and peripheral blood monocytes. Specifically, a CSF-specific macrophage population with an antiinflammatory transcriptomic signature and peripheral CD16^+^ monocytes increased in frequency after B cell depletion. This was accompanied by increases in TNF-α mRNA and protein levels in monocytes following B cell depletion, consistent with the finding that anti–TNF-α treatment exacerbated autoimmune activity in MS. In parallel, B cell depletion induced changes in peripheral CD4^+^ T cell populations, including increases in the frequency of TIGIT^+^ Tregs and marked decreases in the frequency of myelin peptide–loaded, tetramer-binding CD4^+^ T cells. Collectively, this study provides an exhaustive transcriptomic map of immunological changes, revealing different cell-type–specific reprogramming as a result of B cell depletion treatment of MS.

## Abstract 2

We explore how B cell depleting therapies modulate the immune landscape using single-cell RNA sequencing (scRNAseq)

## Introduction

While B cell depletion is efficacious in the treatment of various autoimmune diseases including rheumatoid arthritis and type 1 diabetes (1, 2), it has conferred remarkable therapeutic benefits in early relapsing-remitting multiple sclerosis (MS) (3). The therapeutic benefits suggest a critical role of B cells in MS pathophysiology. While pathogenic myelin-reactive T cells (4, 5) with loss of Treg function (6–8) have been established as mechanistic drivers of MS pathogenesis, accumulating evidence also directly implicates B cells as key contributors to the loss of immune tolerance. The central role of B cells in MS pathophysiology is substantiated by the infiltration of B cells into MS lesions and CSF (9), the presence of ectopic meningeal B cell follicles adjacent to areas of focal cortical demyelination (10, 11), and the detection of oligoclonal IgG bands in the CSF as a diagnostic marker (12, 13).

Recent studies have investigated the effects of B cell depletion therapy with a focus on neuronal cell types and T cells, as well as the characterization of B cells before and after therapy (14–19). In this study, we conducted an unbiased exploration of how B cell–depleting therapies modulate the immune landscape through the use of single-cell RNA-Seq (scRNA-Seq). B cell depletion in patients was accomplished through the humanized anti-CD20 antibody ocrelizumab. We performed 5′ scRNA-Seq on 18 paired PBMC samples and 5 paired CSF samples obtained from patients with incident MS before and after B cell depletion treatment, followed by flow cytometry validation of protein expression. The high-dimensional single-cell dataset allowed for simultaneous interrogation of the diverse immune cell populations in patient blood and CSF and across disease states and treatment statuses.

Our data revealed an increased frequency of a CSF macrophage population after B cell depletion that was paralleled by an increased frequency of CD16^+^ monocytes in the periphery. We discovered that CD16^+^ monocytes had the highest upregulation of transcriptomic TNF-α/NF-κB signatures after ocrelizumab treatment compared with other cell types. Increased TNF-α protein expression in monocytes confirmed these transcriptional changes. Moreover, we found that changes in the myeloid compartment were accompanied by increased T cell immunoreceptor with Ig and ITIM domains–expressing (TIGIT-expressing) FoxP3^+^ Tregs and decreased frequency of circulating myelin-reactive CD4^+^ T cells. Our study provides an extensive transcriptomic map of immunological changes through simultaneous interrogation of the diverse immune cell populations and their transcriptomic states before and after in vivo B cell depletion. This approach enabled us to discover distinct cell-type–specific reprogramming associated with B cell depletion therapy in MS.

## Results

### CD14^+^CD68^+^ CSF cells increase in frequency after B cell depletion therapy.

**Figure 1:**
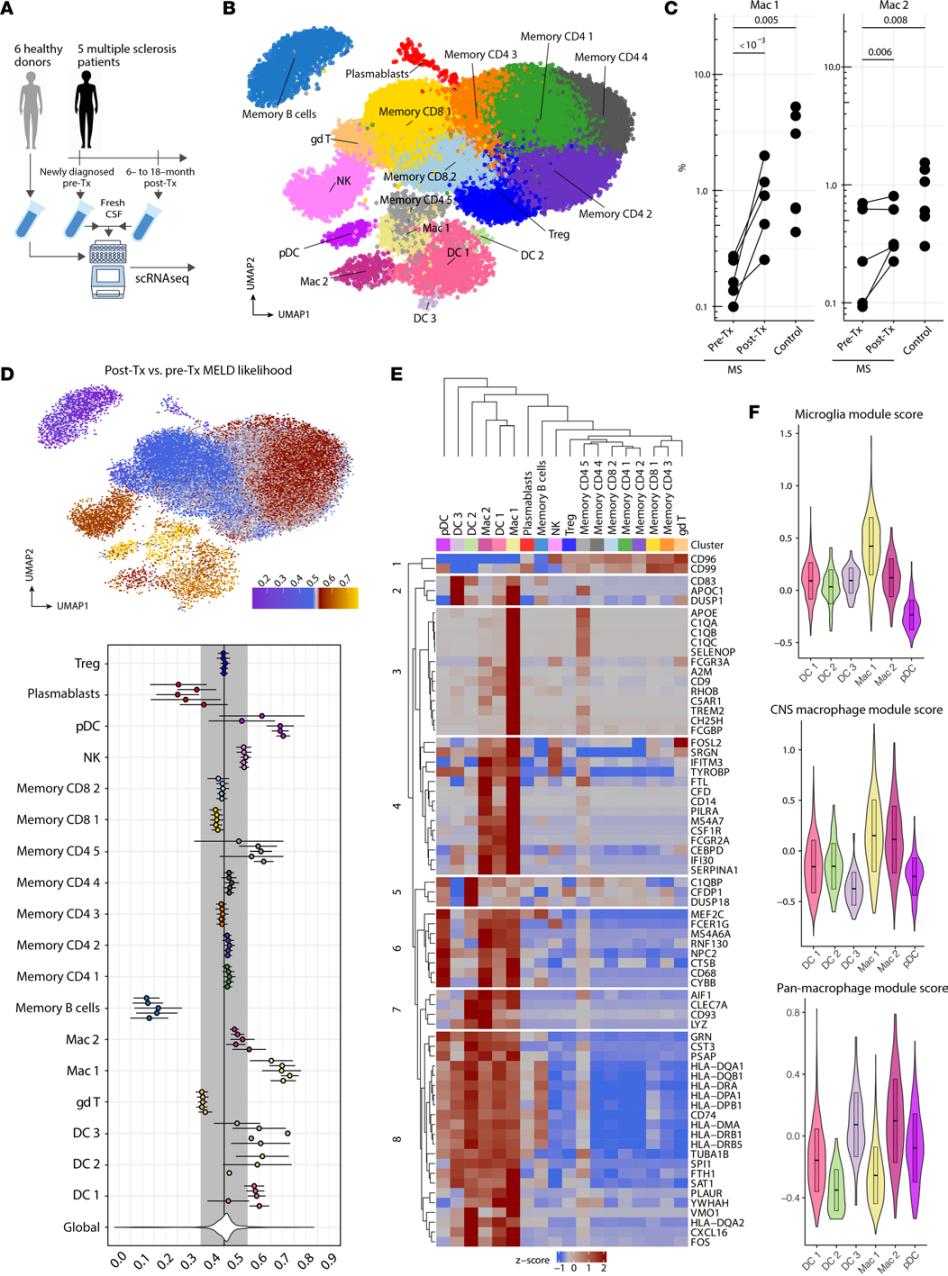
Microglia-like CSF macrophages increase in frequency in patients with MS following B cell depletion therapy. (**A**) Healthy donor and MS patient sample collection scheme for scRNA-Seq analysis (*n* = 6 healthy donors, *n* = 5 patients with MS before treatment [Tx], *n* = 5 matched patients with MS after treatment). (**B**) UMAP dimensionality reduction plot of immune cell clusters detected in CSF from healthy donors and patients with MS (*n* = 60,704 single cells, 17 immune cell clusters). (**C**) CSF macrophage cluster frequency before and after B cell depletion therapy across all 5 patients with MS. (**D**) Post–B cell depletion therapy MELD likelihood (mean ± SEM, *n* = 5 patients with MS) enrichment UMAP and patient-level summary values for all immune clusters in **C**. (**E**) Heatmap of myeloid-related genes showing average expression levels across all immune cell types. (**F**) All myeloid clusters in the CSF scored against microglia, CNS macrophage, and pan-macrophage gene modules. *P* values were calculated using the Wald test of regression coefficients.

All patients had new-onset relapsing-remitting MS and were treatment naive to any immunomodulatory therapy (Supplemental Table 1; supplemental material available online with this article; https://doi.org/10.1172/JCI182790DS1). To elucidate the effects of anti-CD20 treatment on the CNS microenvironment, we analyzed 10 patient-matched fresh CSF samples from 5 patients with RRMS before and after B cell depletion treatment and performed an additional comparison with 6 fresh CSF samples from age-matched healthy donors. Post-treatment CSF samples were obtained at different time points: 2 samples 6 month after treatment, 2 samples 12 months after treatment, and 1 sample 18 months after treatment (Supplemental Table 1). After filtering out low-quality cells, scRNA-Seq yielded a total of 60,704 single cells from 16 CSF samples, including 15,122 cells from 6 healthy donor samples, 28,493 cells from 5 treatment-naive MS samples, and 17,089 cells from 5 post-treatment MS samples (Figure 1A).

After normalization and Harmony batch correction (see Methods), CSF cells were classified into 17 clusters (Figure 1B). To assess potential donor and sample variability, we profiled the frequencies of all immune cell populations in the CSF for each patient. Across all 5 patients, the pre-treatment MS samples had lower proportions of the CD14^+^CD68^+^ myeloid 1 (Mac 1) cluster than did samples from healthy donors, and the frequency of this cluster subsequently increased in all patients after B cell depletion treatment (Figure 1C, Supplemental Figure 1, and Supplemental Data 1). In parallel, in all 5 patients, 1,536 B cells were detected before treatment, and only 188 B cells were detected after treatment, demonstrating effective B cell depletion by anti-CD20 antibodies. To further identify the immune subset most affected by B cell depletion therapy, we used manifold enhancement of latent dimensions (MELD) (20) to quantify the effect of B cell depletion treatment on all immune cell clusters in the CSF and discovered that the same Mac 1 cluster was the most enriched cell type after treatment (Figure 1D).

DC clusters DC 1, DC 2, DC 3, and plasmacytoid DCs (pDCs) also showed a post-treatment enriched MELD score. CSF scRNA-Seq data yielded 1,423 cells in DC 1, 40 cells in DC 2, 35 cells in DC 3, and 256 cells in pDC from all patients across all time points. Given the low total cell numbers in DC 2 and DC 3 clusters, we focused our DC analyses on DC 1 and pDC clusters. The DC 1 cluster showed signatures of type-2 conventional dendritic cells with 440 healthy donor cells, 485 pre-treatment cells, and 498 post-treatment cells. The pDC cluster had 37 healthy donor cells, 88 pre-treatment cells, and 131 post-treatment cells. The DC 1 and pDC clusters showed no differences in MHC class I and class II expression before and after B cell depletion (Supplemental Figure 2A and Supplemental Figure 3A), and DC 1 showed no difference in expression of the antigen presentation genes *CD74* and *CD86* before or after treatment (Supplemental Figure 2B). We also profiled inflammatory genes in the DC 1 and pDC clusters and observed that these genes were expressed in less than 5% of cells across all time points, with the exception of *CD81* and *CD86*, which were expressed in approximately 10%–15% of pDC cells. Notably, CD40 expression was downregulated after B cell depletion in both DC 1 and pDC clusters, suggesting a potential decrease in DC activation and CD40-CD40L cellular interaction with B and T cells (Supplemental Figure 2C and Supplemental Figure 3B). In summary, B cell depletion did not appear to affect the expression of antigen presentation genes in DC 1 or pDC clusters, and DCs in the CSF expressed low transcript levels. Therefore, while the DC clusters exhibited increased trends of post-treatment frequencies similar to those observed in the Mac 1 cluster, we prioritized downstream analyses of the Mac 1 subset, as it had the highest MELD enrichment score and the lowest MELD score variability across patients.

### CD14^+^CD68^+^ CSF cells are CSF-specific macrophages with microglial gene signatures.

Previous studies reported CD14^+^ CSF cells in various disease settings (21–23). We aimed to further evaluate the myeloid transcriptomic signatures in our patient CSF samples to define the CD14^+^CD68^+^ myeloid clusters. Mac 1 clusters exhibited high levels of myeloid (*TREM2*, *SPI1*, *CD68*, *MEF2C*) and monocyte (*CD14, FCGR3A, CSF1R, HLA-DRA*) transcriptomic signatures, while lacking expression of hallmark microglial genes such as *SALL1*, *P2RY12*, *FCRLS*, and *TMEM119* (24, 25). In addition to pan-macrophage lineage markers *HLA-DR* and *CD14*, the Mac 1 clusters also had high expression of *APOE*, *CSF1R*, and genes that are expressed in extraparenchymal CSF macrophages such as *CST*, *TGFB1*, *MS4A7*, *LYZ*, and *CLEC7A*. The mixture of microglia-like and monocytic genes and the abundant expression of *C1Q* and *HLA* class II genes classified this cluster as CSF-specific macrophages, distinct from monocytes, microglia, and macrophages in other CNS tissues (Figure 1E) (26). Interestingly, memory CD4 cluster 5 had a similar (albeit muted) signature, leading to the initial coclustering of those 2 clusters, despite coming from 2 separate lineages (Supplemental Figure 4, A and B; see also Methods).

To further delineate the CSF macrophage subset, we computed pan-macrophage (*CD44*, *CCR2*, *CD45*, *CD206*, *CD163*, *CD274*, *CD169*, *MYB*), CNS macrophage (*TGFBI*, *MS4A7*, *MS4A6C*, *LYZ2*, *CD163*, *P2RX7*, *CST*, *CLEC7A*), and microglial (*P2Y12R*, *TMEM119*, *TREM2*, *CD115*, *CD172A*, *CD91*, *SPI1*, *FCRLS*, *SALL1*, *HEXB*, *SIGLECH*, *SLC2A5*) subset–specific gene module scores on all myeloid clusters in the CSF. The average expression of each of the transcriptomic programs was calculated and then subtracted by the aggregated expression of randomly selected control genes using the AddModuleScore function from the Seurat package (27). The post-treatment enriched Mac 1 cluster scored the highest on the microglia module compared with other myeloid clusters. In contrast, the CD14^+^CD68^+^ myeloid 2 (Mac 2) cluster scored higher on the pan-macrophage module compared with the others (Figure 1F). Collectively, these transcriptomic signatures reflect the phenotypic diversity of the macrophage compartment in the CSF and show that anti-CD20 treatment increased the frequency of a specific subset of CSF macrophages within the CSF.

### B cell depletion is associated with an antiinflammatory phenotype in CSF macrophages.

**Figure 2:**
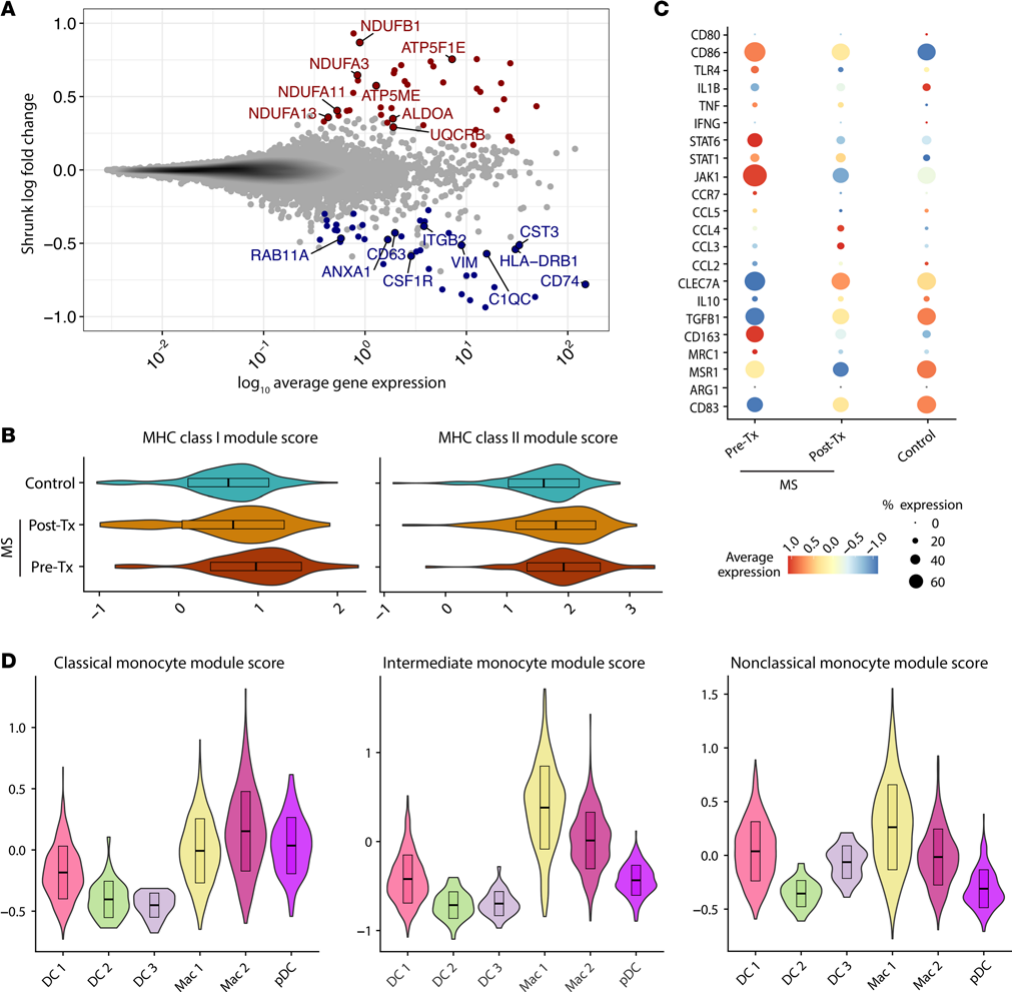
Enriched CSF macrophages present an antiinflammatory phenotype in patients with MS following B cell depletion therapy. Gene expression analyses of CSF macrophages were conducted by comparing cells from healthy donors (*n* = 6) and pre– and post–B cell depletion therapy MS patient samples (*n* = 5). (**A**) Mean abundance plot depicting DEGs (FDR <0.1) in the Mac 1 cluster from patients with MS before and after B cell depletion. Blue indicates downregulation after treatment; red indicates upregulation after treatment. (**B**) MHC class I and class II gene module scores for healthy donor, MS pre-treatment, and MS post-treatment Mac 1 cells. (**C**) Dot plot depicting myeloid inflammatory and antiinflammatory gene expression in healthy donors and patients with MS before and after treatment. (**D**) Gene module scores for all CSF myeloid clusters against peripheral monocyte gene signatures. Top: classical monocyte module score, middle: intermediate monocyte module score; bottom: nonclassical monocyte module score.

With the recent identification of CSF-specific macrophages (26) and the limited availability of patient CSF samples, the role of CSF macrophages in homeostasis and during MS pathogenesis remains unclear. To better understand the treatment effect of B cell depletion on the CSF macrophage immunophenotype, we performed analysis of differentially expressed genes (DEGs) in pre- and post-treatment MS samples along with healthy donor CSF (Figure 2A and Supplemental Data 2). In the enriched Mac 1 cluster, hierarchical clustering on DEGs in pre-treatment MS cells versus healthy donor cells revealed extensive changes, segregating cells from the 2 groups and highlighting alterations in the CSF macrophages of patients with MS (Supplemental Figure 5A). In contrast, hierarchical clustering between MS pre- and post-treatment CSF macrophages resulted in limited separation between the 2 groups (Supplemental Figure 5B). Among the genes upregulated after B cell depletion were oxidative phosphorylation genes (genes coding for NADH dehydrogenase and ATP synthase subunits) that are associated with antiinflammatory macrophages (28, 29) (Figure 2A). In addition, genes involved in migration and endocytosis (*ITGB2*, *CSF1R*, *RAB11A*, *ANXA1*), as well as *HLA* class II genes, were downregulated after B cell depletion.

We observed increases in *HLA* class I and class II mRNA expression in Mac 1 cells from MS patient pre-treatment CSF as compared with healthy CSF Mac 1 cells. These increases in MHC expression from patients with MS decreased after B cell depletion (Figure 2B). We next applied the classical and alternative macrophage activation paradigm to delineate the neuroinflammatory state of CSF macrophages. Post-treatment Mac 1 cells exhibited transcriptomic downregulation of proinflammatory programming genes (*CCR7, JAK1, STAT1, IL1B, TNFA, TLR4, CD86*) and upregulation of antiinflammatory genes (*IL10, TGFB, CLEC7A*). In addition, we observed decreased expression of macrophage scavenger receptor genes (*CD163, MRC1, MSR1*) (Figure 2C). Taken together, the decreases in MHC class I and class II gene module scores suggest that B cell depletion reduced the probability of T cell activation through CSF macrophage antigen presentation. The increased expression of *IL10*, *TGFB*, and the oxidative phosphorylation pathway genes in post-treatment CSF macrophages suggests that B cell depletion therapy promoted the resolution of the inflammatory phenotype in CSF macrophages to restore homeostasis. Last, we computed transcriptomic signature scores on the basis of peripheral monocytic gene modules and applied them to the CSF myeloid cell populations. We found that the enriched Mac 1 cluster scored the highest on the intermediate monocyte (*HLA-DR*, *CD14*, *CD11C*, *CD68*, *FCGR3A*, *CX3CR1*, *CSF1R*, *TLR4*) and nonclassical monocyte (*FCGR3A*, *CX3CR1*, *SLAN*, *CSF1R*, *CXCR1*, *CXCR4*) gene modules, whereas Mac 2 scored higher on the classical monocyte (*CD14*, *CCR2*, *CCR5*, *SELL*, *CD36*, *CD33*, *CD64*) gene module (Figure 2D). Thus, the transcriptomic resemblance of CSF macrophages to intermediate monocytes prompted us to investigate whether B cell depletion therapy modulates intermediate monocyte frequency in the periphery.

### Increased abundance of CD16^+^ monocytes after B cell depletion in PBMCs from patients with MS.

**Figure 3:**
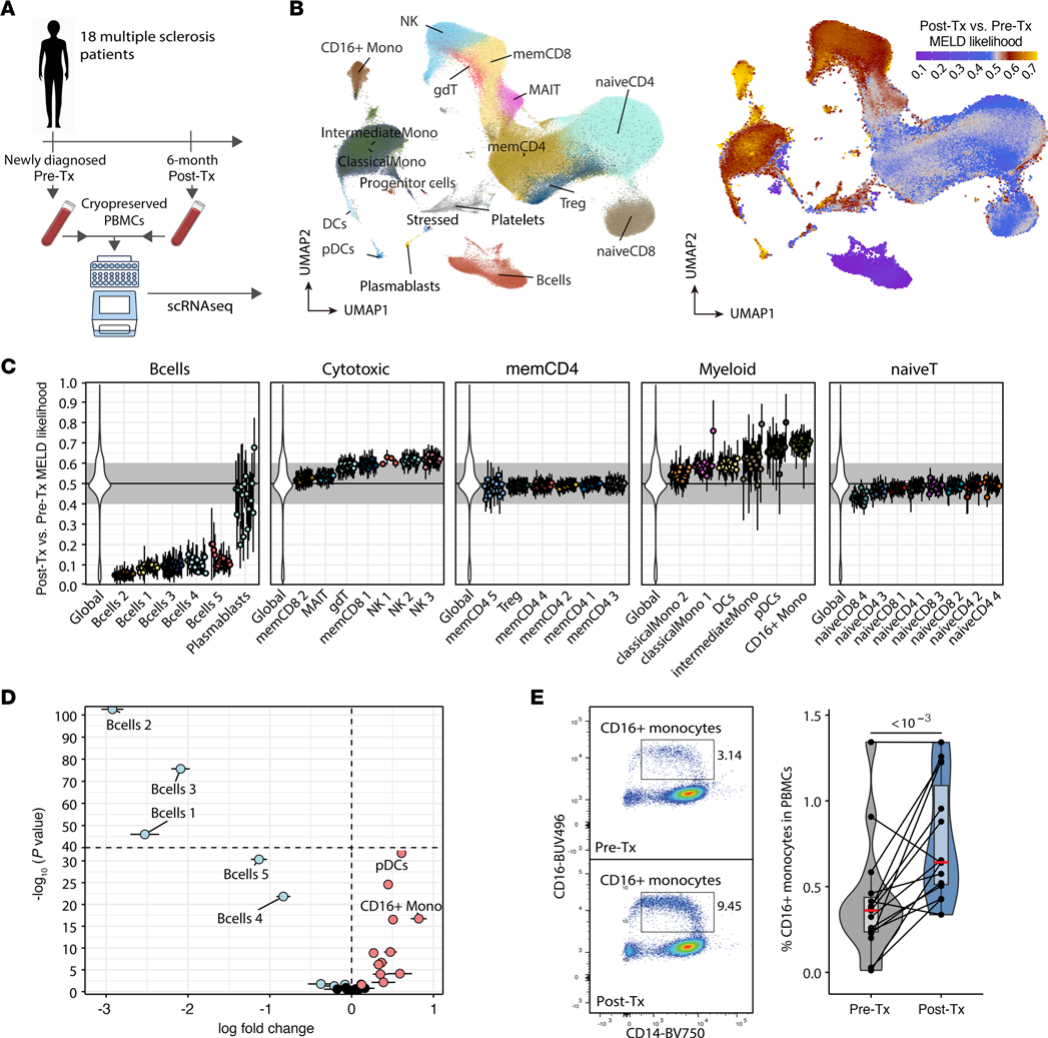
Increased CD16^+^ monocyte abundance after anti-CD20 treatment. (**A**) experimental design of pre- and post-treatment (anti-CD20) PBMC samples from patients with MS (*n* = 18) for droplet-based scRNA-Seq using the 10X Genomics platform. (**B**) UMAP of annotated cell types (left) and overlaid MELD likelihoods for post-treatment status (right). (**C**) MELD likelihood patient-level summary values (mean ± SEM) per fine-grained cluster and main cell types. (**D**) Fine-grained community frequency post-treatment changes (log fold change mean estimate ± SEM from β regression; see Methods). (**E**) Flow cytometric validation of CD16^+^ monocyte frequency changes in MS patients’ PBMCs. *P* values were calculated using the Wald test of regression coefficients.

We next investigated whether alterations observed in the CSF after B cell depletion were recapitulated in peripheral blood. We performed immune profiling with cryopreserved PBMCs using scRNA-Seq from 18 newly diagnosed treatment-naive patients with RRMS, whose samples were collected both before and 6 months after treatment (Supplemental Table 1 and Figure 3A). In our unsupervised analysis, we retained 38 communities, which we assigned to coarse-grained immune cell types of interest (naive T cells, memory CD4^+^ T cells, cytotoxic lymphocytes, B cells, myeloid cells) and 18 fine-grained cell types, as described in Figure 1B and Supplemental Figure 6, A and B. We classified communities into main lineages on the basis of marker gene inspection and scored cells against reference datasets using singleR software (Supplemental Figure 6C) (30).

Using MELD, we assessed differences in abundance between the pre- and post-treatment samples. As expected, we found that ocrelizumab treatment substantially decreased the abundance of B cells except for plasmablasts, which are known to downregulate CD20, leading to loss of sensitivity to ocrelizumab-mediated depletion (Figure 3B). Given our CSF data, we next focused our attention on myeloid cells. We observed cellular enrichment in 2 myeloid clusters: CD16^+^ monocytes and pDCs (Figure 3C). We confirmed these changes by formally testing for variations in frequency across all donors and observed that the increased frequency of CD16^+^ monocytes was conserved across donors, whereas variations in pDC frequencies were more heterogenous (Figure 3D, Supplemental Figure 7A, and Supplemental Data 3). This led us to focus solely on CD16^+^ monocytes for further analyses. The CD16^+^ monocyte cluster was the only cluster with detectable levels of *FCGR3A*, the gene encoding CD16 (Supplemental Figure 7, B and D), and displayed markers associated with intermediate and nonclassical monocytes. Scoring against the Monaco Immune reference with singleR revealed strong enrichment in intermediate and nonclassical reference transcriptomes (Supplemental Figure 7C). To confirm this change, we measured circulating frequencies of various monocyte subpopulations using flow cytometry (Supplemental Figure 8A). We noted a significant increase in CD14^+^CD16^+^ monocytes after B cell depletion treatment (*P* < 1 × 10-3 ) (Figure 3E).

### Increased activation and TNF-α production in CD16^+^ monocytes following B cell depletion.

**Figure 4:**
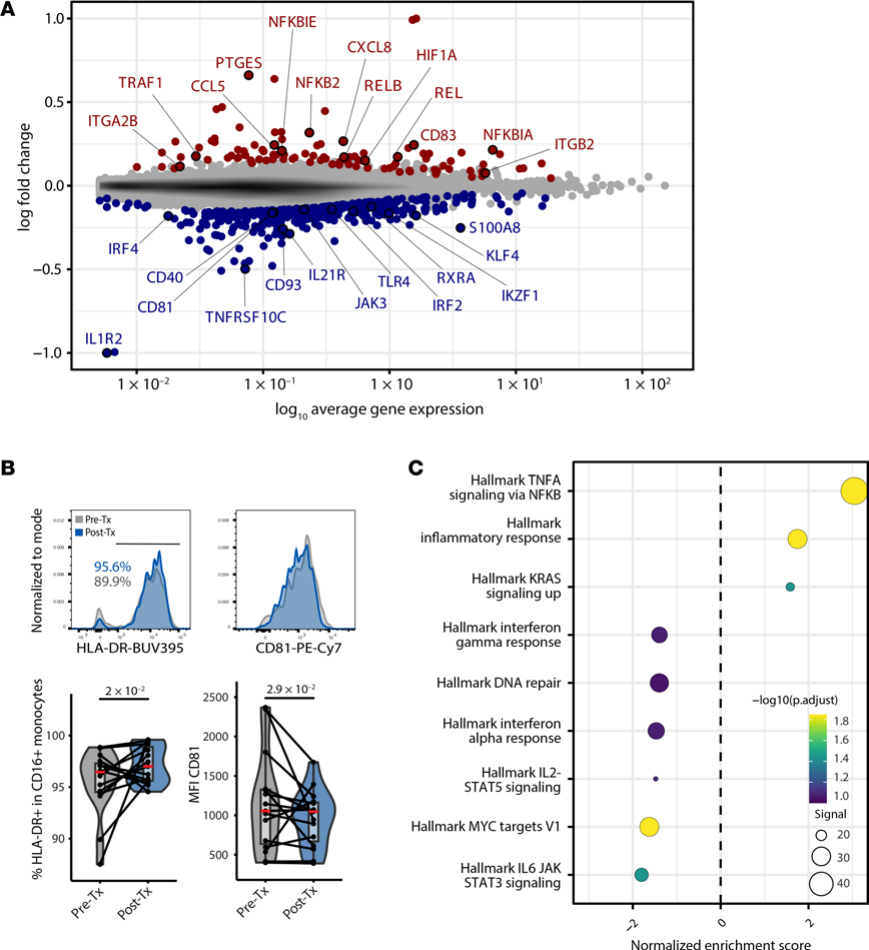
Post-treatment DEGs in CD16^+^ monocytes. (**A**) Mean abundance plot of gene expression changes. DEGs are highlighted in red (upregulated after treatment), or blue (downregulated after treatment). (**B**) Flow cytometric analysis of HLA-DR and CD81 expression in CD16^+^ monocytes (*n* = 16). (**C**) GSEA using the Hallmark gene sets for CD16^+^ monocytes. (**D**) Custom GSEA analysis of post-treatment PBMC monocyte signature gene sets (up- and downregulated genes) tested on the CSF macrophage Mac 1 dataset (from Figure 1). *P* values were calculated using the Wald test of regression coefficients.

We next determined whether CD16^+^ monocytes harbor an altered transcriptomic state after B cell depletion. We computed DEGs while controlling for interindividual variation using a generalized linear mixed model (glmm, as implemented in NEBULA) (31) (Supplemental Data 4). We observed increased expression of soluble molecule genes (*CCL5*, *CXCL8*, *TNFA*), surface receptor genes (*CD83*, *ITGB2*, *ITGA2B*), the transcription factor *HIF1A*, and NF-κB signaling pathway genes (*TRAF1*, *NFKB2*, *REL*, *RELB*, *NFKBIA*) (Figure 4A). HIF1A has been shown to play an essential role in promoting antiinflammatory activity in myeloid DCs (32). In addition, CD83, a macrophage immune checkpoint marker, contributes to the resolution of inflammation and can induce Tregs in experimental models of MS (33, 34). CD16^+^ monocytes showed downregulation of select transcription factor genes (*RXRA*, *IKZF1*, *KLF4*, *IRF4*) and CD81, a marker that facilitates monocytes homing to the CNS in experimental autoimmune encephalomyelitis (EAE) (35). Given the transcriptomic signature observed in CD16^+^ monocytes, we validated changes at the protein level and observed downregulation of CD81 and upregulation of the monocyte activation marker *HLA-DR* using flow cytometry (Figure 4B).

To further investigate the DEGs from the NF-κB signaling pathway, we next performed gene set enrichment analysis (GSEA) of the enriched CD16^+^ monocyte cluster using the Hallmark gene set collection. Consistent with the increased expression of NF-κB–relevant transcription factors, we observed enrichment in the TNF-α/NF-κB pathway (Figure 4C), as well as downregulation related to JAK/STAT signaling gene sets (IL-2, STAT5, IL-6, JAK, STAT3, IFN-α, and IFN-γ signaling pathways) after treatment. Finally, to determine whether these pathway modulations can also be observed in CSF macrophages (described in Figure 2), we created custom gene sets based on PBMC differential gene expression and ran GSEA on the enriched Mac 1 CSF macrophages to test for gene signature enrichment. We observed no significant enrichment of PBMC gene sets in Mac 1 CSF macrophages (Supplemental Figure 9), suggesting the treatment-mediated in vivo perturbation of biological pathways was tissue specific and informed by the local environment.

### B cell depletion induces a ubiquitous response to TNF-α in PBMCs.

**Figure 5:**
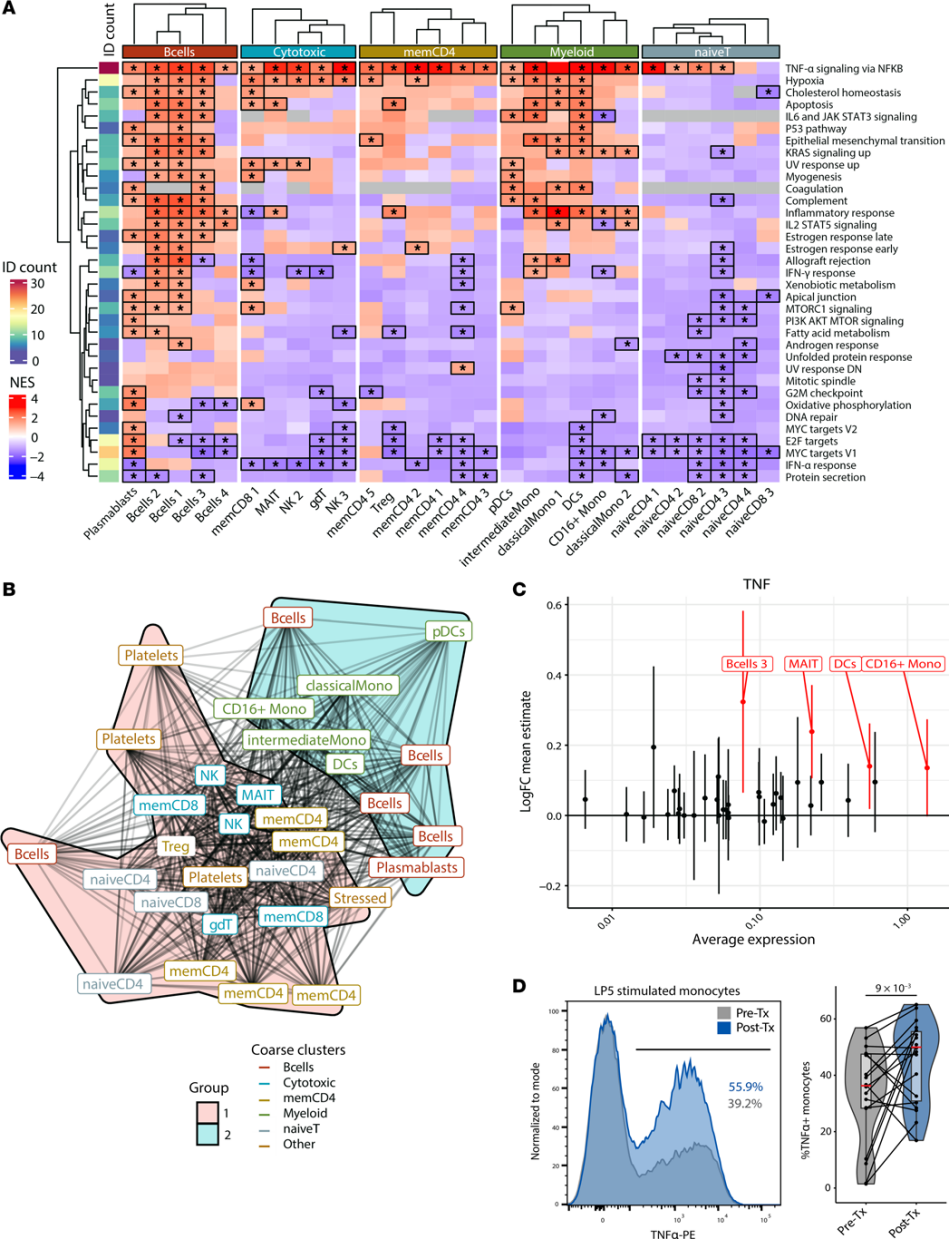
GSEA of anti-CD20 gene expression alterations across cell types. (**A**) Heatmap of normalized enrichment scores (NESs) from post-treatment GSEA analyses run for each cluster shows a ubiquitous increase in TNF-α/NF-κB pathway genesets. Differentially enriched gene sets are highlighted with an asterisk. “ID count” indicates the number of times a gene set was found enriched across communities. (**B**) Overlap graph analysis of leading-edge genes for the “TNF-α signaling via NF-κB” gene set across cell types highlights 2 sets of signatures: B and myeloid cells versus T cells. (**C**) Pre- and post-treatment fold change of *TNFA* transcript levels across clusters (differential expression is highlighted in red). (**D**) In vitro validation of TNF-α upregulation before and after B cell depletion at the protein level in monocytes from patients with MS (*n* = 18) by intracellular flow cytometry staining after LPS stimulation. *P* values were calculated using the Wald test of regression coefficients.

We next assessed whether the observed upregulation of TNF-α/NF-κB pathway after treatment is cell-type specific. We computed DEGs for pre– versus post–B cell depletion in all clusters (Supplemental Data 4) and used differential gene expression results to run GSEA analysis. We observed TNF-α/NF-κB pathway activation across most immune cell types after ocrelizumab treatment (Figure 5A). However, the downregulation of JAK/STAT-related pathways was restricted to CD16^+^ monocytes, and the remaining or repopulating B cells after treatment showed increased expression of various gene sets related to cell survival (P53 pathway, apoptosis), metabolism (cholesterol homeostasis), and JAK/STAT signaling (IL-2/STAT5 and IL-6/JAK/STAT3 pathways). Interestingly, clustering communities based on GSEA leading-edge gene similarity showed that the NF-κB signaling enrichment was most similar between B cells and myeloid cells, whereas T lymphocytes formed a separate cluster, suggesting that transcriptomic responses to NF-κB signaling differed between those lineages (Figure 5B).

Despite the ubiquitous TNF-α/NF-κB pathway activation across cell types, CD16^+^ monocytes showed the highest pre-treatment transcriptomic expression of TNF-α and upregulated expression after treatment (Figure 5C). We also detected increased post-treatment expression of TNF-α in B cell cluster 3, mucosal-associated invariant T cells, and DC clusters. To confirm changes in TNF-α expression at the protein level, we enriched CD14^+^ or CD16^+^ monocytes from cryopreserved PBMCs and showed that LPS-stimulated monocytes from patients with MS expressed more TNF-α following B cell depletion (Figure 5D). Increases in TNF-α production by LPS-stimulated CD14^+^ cells were similarly observed in patients with MS treated with ocrelizumab by another group (36). However, there was no significant difference in TNF-α levels in culture supernatant measured by ELISA (data not shown).

### B cell depletion therapy reprograms the CD4^+^ Th cell compartment in both CSF and PBMCs.

**Figure 6:**
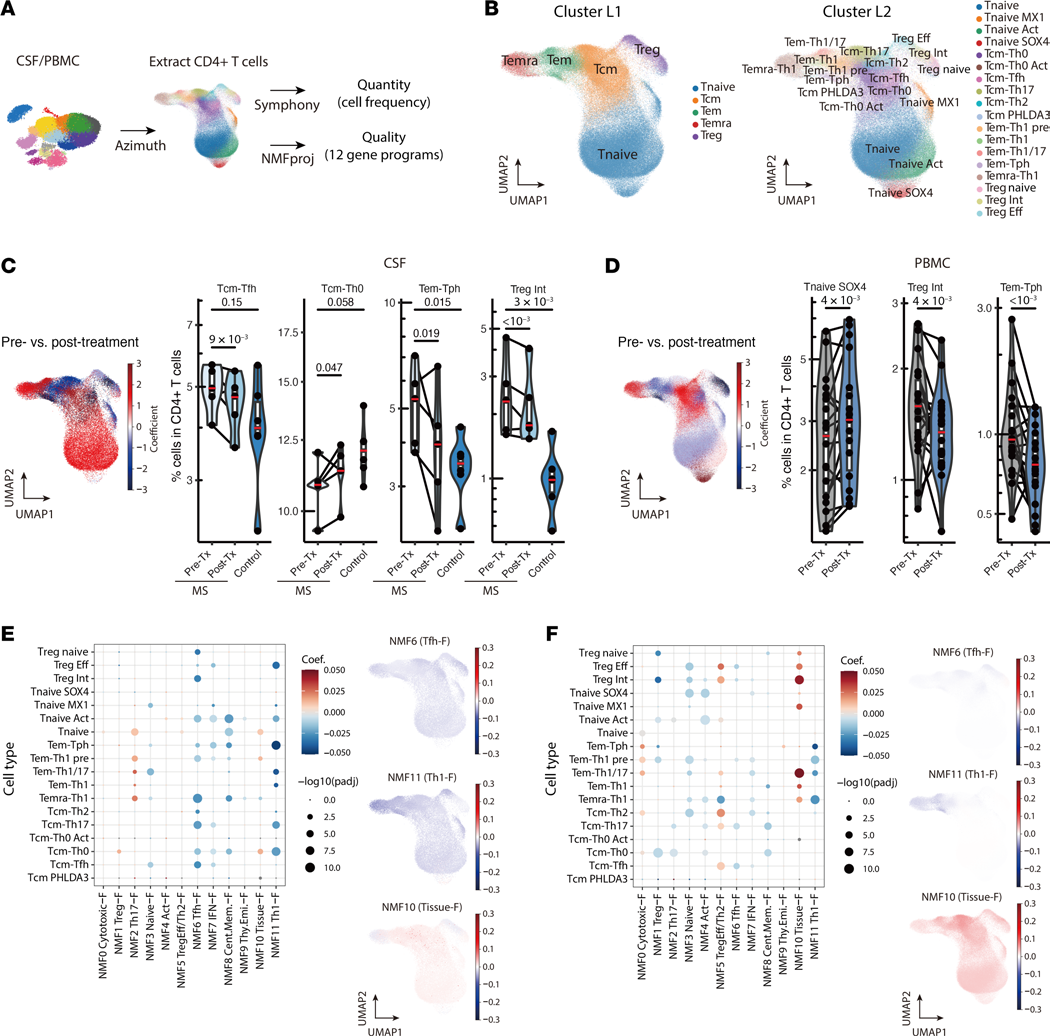
Detailed analysis of CD4^+^ T cell alterations following anti-CD20 treatment. (**A**) Schematic illustration of the analysis of CD4^+^ T cells using reference mapping and NMFproj. From CSF and PBMC samples, CD4^+^ T cells were extracted using Azimuth, and detailed CD4^+^ T clusters were predicted using Symphony. The 12 gene programs were calculated using NMFproj. (**B**) Inferred CD4^+^ T cell clusters on the UMAP plot. The clusters were assigned to either a major cluster (L1) or a detailed cluster (L2) level. (**C** and **D**) Cell frequency changes after anti-CD20 treatment in CSF (**C**) and PBMCs (**D**). Coefficients of cell frequency change per cluster L2 quantified using a generalized linear model with beta distribution are visualized on the UMAP plot (left). The populations with cell frequency increases following B cell depletion treatment are shown in red. CD4^+^ T cluster frequency before and after B cell depletion therapy (right). Substantially altered clusters are shown. See Supplemental Figure 10C and Supplemental Figure 11C for additional details. (**E** and **F**) Alterations of gene programs extracted by NMFproj after anti-CD20 treatment in CSF (**E**) and PBMCs (**F**). Dot plots depicting NMF cell feature changes in each cell type (left). Dot colors show coefficients, and sizes show the significance of GLM (method). The coefficient (Coef.) of the gene program change per cluster for some gene programs is shown on the UMAP plots (right). Annotations and representative genes of gene programs are as follows: NMF0 (cytotoxic-feature [cytotoxic-F]; *GZMB*, *CX3CR1*); NMF1 (Treg-F; *FOXP3*, *IL2RA*); NMF2 (Th17-F; *RORC*, *CCR6*); NMF3 (naive-F; *CCR7*, *BACH2*); NMF4 (activation-F [Act-F]; *DACT1*, *CDK6*), NMF5 (Treg Eff/Th2-F; *HLA-DR*s, *CCR10*); NMF6 (Tfh-F; *MAF*, *CXCR5*); NMF7 (IFN-F; *OAS1*, *MX1*); NMF8 (central memory-F; *CRIP2*, *PLP2*); NMF9 (thymic emigrant-F; *SOX4*, *PECAM1*); NMF10 (tissue-F; *JUNB*, *NFKBIA*); and NMF11 (Th1-F; *GZMK*, *EOMES*). *P* values were calculated using the Wald test of regression coefficients.

Although CD4^+^ T cells have been demonstrated to be key pathogenic drivers of MS pathophysiology, we observed limited changes in the CD4^+^ T cell compartment using singleR, MonacoImmune, and manual cellular annotation methods. Therefore, we applied a recently developed framework that captures a more granular classification and qualitative assessment of CD4^+^ T cells based on scRNA-Seq data (Figure 6A) (37). This framework assigned CD4^+^ T cells to 5 major clusters (cluster layer 1 [L1]) and 18 minor clusters (cluster L2) by Symphony reference mapping (Figure 6B, Supplemental Figure 10A, and Supplemental Figure 11A) and measured the activity of 12 predefined transcriptomic gene programs of CD4^+^ T cells using non-negative matrix factorization projection (NMFproj).

At the major cluster L1 level, we did not detect any significant cell frequency changes in both CSF or PBMCs (Supplemental Figure 10B and Supplemental Figure 11B). However, at the minor cluster L2 level, there was a significant reduction in CD4^+^ T effector memory (Tem) cells expressing T peripheral helper (Tph) markers (Tem-Tph; *PDCD1^lo^CXCR5^+^*) in both CSF (adjusted *P* value [*P*adj] = 1.88 × 10^–2^) and PBMCs (*P*adj = 9.41 × 10^–6^) tissues after B cell depletion treatment (Figure 6, C and D, Supplemental Figure 10C, and Supplemental Figure 11C). In CSF alone, the frequency of CD4^+^ T central memory (Tcm) expressing T follicular helper (Tfh) markers (Tcm-Tfh; *PDCD1*^+^*CXCR5*^+^) was significantly reduced (*P*adj = 9.36 × 10^–3^), while the frequency of Tcm-Th0 was increased (*P*adj = 0.0472), suggesting a shift toward a naive phenotype following B cell depletion in the CNS (Figure 6C and Supplemental Figures 10C). We also observed a significant increase in CD4^+^ naive T cells expressing *SOX4* (Tnaive *SOX4*; *SOX4^+^PECAM1^+^*) (*P*adj = 4.29 × 10^–3^) in the blood, a recent thymic emigrant cell population (38), indicating that the peripheral blood CD4^+^ T cell pool had been replenished by newly generated CD4^+^ T cells after treatment (Figure 6D and Supplemental Figure 11C).

We then assessed the changes in gene program activity quantified by NMFproj in each L2 subpopulation. We observed a significant reduction in cell types for NMF6 (Tfh-feature [Tfh-F]; *MAF*, *CXCR5*) and NMF11 (Th1-F; *GZMK*, *EOMES*) post-treatment in both blood and CSF. (Figure 6, E and F, Supplemental Data 5, and Supplemental Data 6). Because NMF6 (Tfh-F) is predominantly high in Tcm-Tfh, Tem-Tph, and intermediate Tregs (Treg Int), the decrease in NMF6 (Tfh-F) in both tissues indicates that B cell depletion treatment reduced their frequencies and potentially repressed the repopulation of these CD4 subtypes quantitatively and qualitatively. An increase in NMF2 (Th17-F; *RORC*, *CCR6*) in the Tem population was observed in CSF, whereas the NMF2 signature decreased in the blood (Figure 6, E and F, Supplemental Data 5, and Supplemental Data 6). We also observed increased NMF10 (tissue-F; *JUNB*, *NFKBIA*) in blood after treatment (Figure 6F and Supplemental Data 6). These observations suggested a redistribution of the CD4^+^ T subsets between the periphery and CNS. Altogether, these findings demonstrate that B cell depletion notably altered the CD4^+^ T cell compartment by reducing specific T cell populations such as Treg Int, Tcm-Tfh, and Tem-Tph and modifying effector gene expression profiles such as repression of NMF6 (Tfh-F) and NMF11 (Th1-F), which may be associated with the therapeutic efficacy of B cell depletion in MS.

### B cell depletion increases Treg frequencies and suppressive phenotypes.

**Figure 7:**
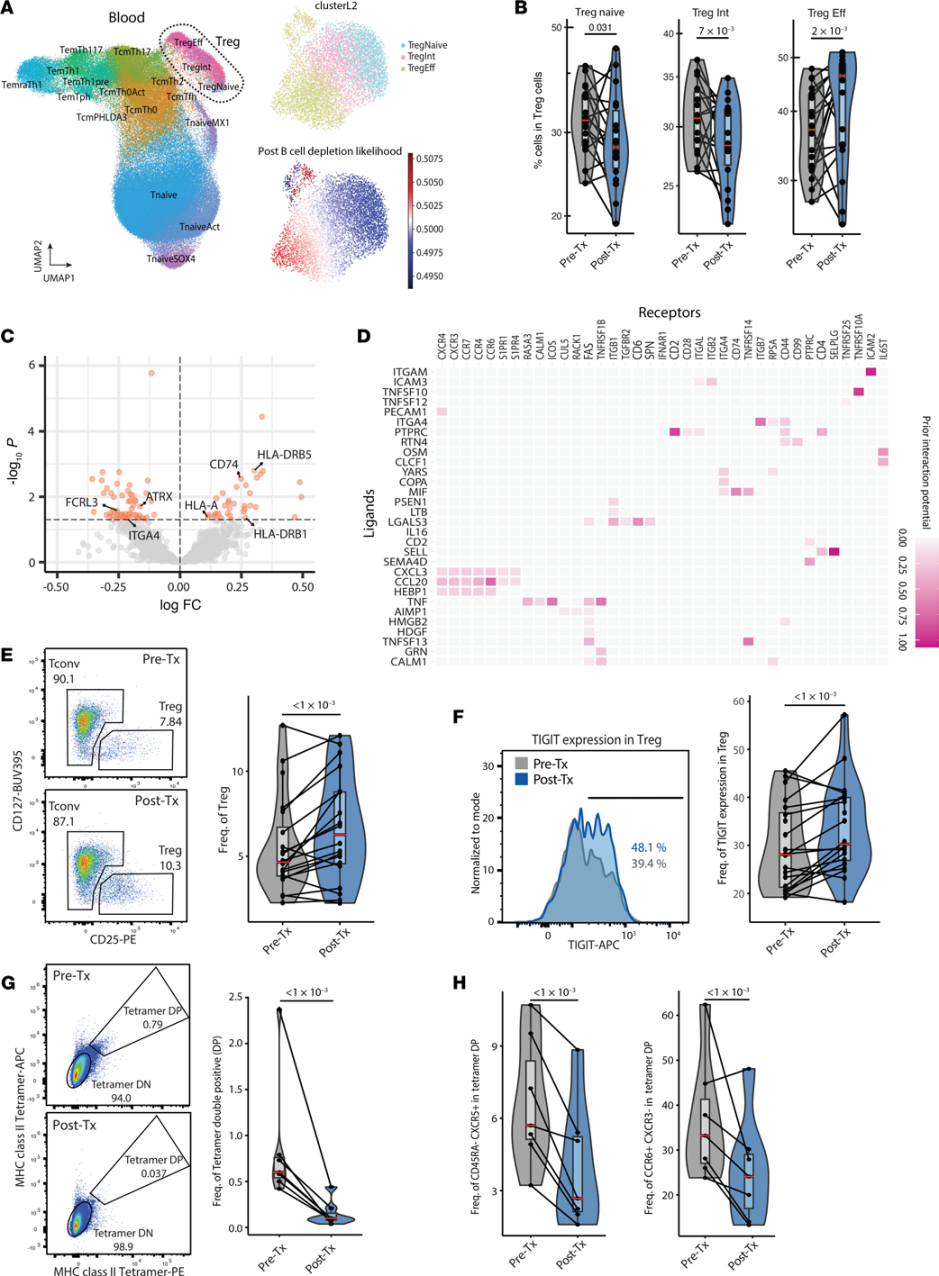
B cell depletion induces an increase in TIGIT^+^ Tregs and reduces autoreactive T cells. (**A**) Visualization of Treg population extraction and changes after B cell depletion treatment. Predicted CD4^+^ T clusters and Tregs (outlined by dotted line) on UMAP plot (left). Re-embedding of extracted Tregs using UMAP (top right). B cell depletion treatment–associated relative likelihood in Treg populations calculated using MELD (bottom right). (**B**) Frequency changes of each subpopulation within the Treg group. (**C**) Volcano plot depicting DEGs in Tregs, particularly highlighting genes encoding surface proteins. (**D**) Heatmap displaying predicted interactions between myeloid cell–derived ligands (limited to genes differentially regulated with B cell depletion treatment) and Treg-derived receptors, weighted by prior interaction potential. (**E** and **F**) Flow cytometric data of Treg frequencies (**E**) and TIGIT protein expression by Tregs (**F**) in MS patient PBMCs (*n* = 20) after B cell depletion treatment. (**G**) Flow cytometric analysis of myelin tetramer–reactive CD4^+^ T cell frequencies (Freq.) at the pre-treatment and 6-month post-treatment time points (*n* = 7). (**H**) Cell frequencies of Tfh (CD45RA^–^CXCR5^+^) and Th17 (CCR6^+^CXCR3^–^) in tetramer-reactive CD4^+^ T cells at pre-treatment and 6-month post-treatment time points (*n* = 7)”. *P* values were calculated using the Wald test of regression coefficients.

Loss of Treg function has been repeatedly observed in patients with MS (8, 39). Our group showed that the MS susceptibility variant can modulate Treg function (40), and Treg function could be compromised during chronic inflammation (6, 41). We subclustered Tregs from scRNA-Seq PBMC data and compared L2 subpopulation frequencies to examine Treg alterations in more detail. We observed a significant decrease in naive Tregs (*FOXP3*, *CCR7*, *P*adj = 3.13 × 10^–2^) and Treg Int (*FOXP3*, *FCRL3*, *P*adj = 6.79 × 10^–3^) and an increase in effector Tregs (Treg Eff) (*HLA-DR*s, *CD74*, *P*adj = 2.33 × 10^–3^) in post-treatment samples. (Figure 7, A and B). We also examined gene expression differences in the whole Treg population and found that *HLA-DR*s and *CD74*, which are the markers of Treg Eff cells, were increased. In contrast, *FCRL3*, a marker of Treg Int, was decreased after treatment (Figure 7C and Supplemental Figure 12, A and B). These data suggest that B cell depletion skewed the Treg function toward the effector phenotype. Next, we examined the potential mediators of myeloid cell–Treg interactions using ligand-receptor prediction analysis (Figure 7D). Here, we observed a potential interaction between TNF receptor 2 (*TNFR2*, encoded by TNFRSF1B) on Tregs and TNF produced by myeloid cells.

Since TNF-α is known to enhance the function of Tregs through TNFR2, which is preferentially expressed in Tregs (42, 43), we hypothesized that monocytes upregulate *TNFA* expression following B cell depletion and promote Treg expansion through TNFR2 signaling. We performed flow cytometry to measure Treg frequencies in 20 MS patient–matched PBMC samples before and after ocrelizumab treatment. Among the 20 samples, 11 were from patients analyzed in our PBMC scRNA-Seq data, and 9 samples were from an additional cohort. We observed a significant increase in Treg frequency (*P* < 0.001) in the post-treatment PBMC samples (Figure 7E). TIGIT protein is highly expressed in Treg Eff cells (44) (Supplemental Figure 12, B and C) and has been shown to associate with increases in functional activity in humans and mice (45, 46). Therefore, we measured the frequency of TIGIT-expressing Tregs after B cell depletion and observed a significant increase after ocrelizumab treatment (*P* < 1 × 10^-3^) (Figure 7F). In summary, these data indicate that B cell depletion was associated with increased Treg frequency and effector function.

### B cell depletion decreases myelin tetramer–binding CD4^+^ T cells.

We and others have shown increases in the frequency of inflammatory myelin–reactive T cells recognizing a number of myelin antigens, presumably as a consequence of epitope spreading, in the circulation of patients with MS. We have demonstrated the effectiveness of using a panel of MHC class II (DRB1*15:01 or DRB1*04:01) tetramers loaded with myelin epitopes from myelin basic protein (MBP), proteolipid protein (PLP), and myelin oligodendrocyte glycoprotein (MOG) to study autoreactive T cells in MS (4, 5, 47, 48). To increase the accuracy of detecting autoreactive T cells, we used both phycoerythrin-conjugated (PE-conjugated) and allophycocyanin-conjugated (APC-conjugated) tetramers with the same myelin composition (Supplemental Figure 8B) and MHC class II–associated invariant chain peptide (CLIP) tetramers as a negative control (Supplemental Figure 8C). We initially examined a cohort of 7 patients with HLA types of DRB1*15:01 and/or DRB1*04:01 before and after B cell depletion and observed a marked decrease in the frequency of myelin PE and APC tetramer–double-positive (tetramer DP) CD4^+^ T cells (Figure 7G). In these data, we detected decreased frequencies of CD45RA^–^CXCR5^+^ cells and CCR6^+^CXCR3^–^ cells in myelin tetramer–binding CD4^+^ T cells (Figure 7H). In a validation cohort of 9 patients with MS, the frequency of myelin-specific T cells was significantly decreased after ocrelizumab treatment, and the frequencies of CD45RA^–^CXCR5^+^ and CCR6^+^CXCR3^–^ cells were also decreased in autoreactive CD4^+^ T cells (Supplemental Figure 13, A and B). Longitudinal kinetics analysis using fresh PBMCs derived from a separate MS patient cohort (Supplemental Table 1) revealed substantial decreases in the frequencies of these autoreactive CCR6^+^CXCR3^+^CD4^+^ T cells 6 months after treatment, whereas no significant changes were observed in CCR6^+^CXCR3^+^ cells in non-tetramer-reactive CD4^+^ T cells. This trend continued through 52 weeks (Supplemental Figure 13C). These results indicate that B cell depletion might regulate the frequency of pathogenic myelin-reactive T cells.

## Discussion

This study provides what we believe to be the first transcriptomic profiling dataset of MS patient blood and CSF pre– and post–B cell depletion therapy. Ocrelizumab, a humanized anti-CD20 monoclonal antibody, induces systematic removal of naive and memory B cells, with approximately 98% efficacy in preventing new CNS lesions (3). Our unbiased analysis of the immune landscape using scRNA-Seq revealed that B cell depletion increased the frequencies of CSF macrophages and that these increased cell populations appeared to exhibit an antiinflammatory phenotype. In the periphery, CD16^+^ monocytes showed the highest level of *TNFA* mRNA expression before treatment, and B cell depletion further increased TNF-α protein expression in these monocytes. Furthermore, after B cell depletion, we observed shifts in T cell populations, including decreased frequencies of Tem-Tph and Treg Int, along with Th1- and Tfh-type gene programs. Notably, we found that B cell depletion increased TIGIT^+^ Treg frequency and decreased myelin tetramer-binding CD4^+^ T cell frequency in the blood compartment.

The changes in myeloid cell frequency after B cell depletion were unexpected and revealed the power of an unbiased systems approach in dissecting the multitudes of immune-regulatory mechanisms of a highly efficacious therapy. We hypothesize that the dichotomous roles of myeloid cells in the pathogenesis of MS are due to the manner in which myeloid phenotype and function are orchestrated by the metabolic requirements in surrounding tissue environments. Soluble factors and cytokines shape macrophage differentiation and activate molecular programs that either exacerbate or attenuate disease (49). Systemic depletion of pathogenic B cells likely reduces the inflammatory states of multiple immune cell types in a feedback loop manner, and the subsequent recalibration of the cytokine milieu allows macrophages to differentiate in a steady-state environment and engage in a disease-resolving transcription program to restore homeostasis. Given that our study is limited to 1 post-treatment time point, it is possible that the changes we describe will normalize over the long term. Our ability to detect functional changes in the CSF compartment was limited by the small sample size, the variable post-treatment time points, and the small number of cells within the CSF macrophages clusters compared with other immune cell populations. However, previous studies dissected changes in myeloid populations in the EAE model and observed CNS-specific alterations of macrophage and microglial phenotypes during the disease course (24, 25). Interestingly, Mrdjen et al. noted that border-associated macrophages (BAMs) share similar profiles with pDCs versus other myeloid subsets. We similarly observed that an increased abundance of CSF macrophages was accompanied by an increased abundance of pDCs (Figure 1). Finally, it should be noted that the increases in frequency of CSF myeloid cells (Mac 1) were unlikely due to a reduction in lymphocyte counts as the absolute number of myeloid cells and lymphocytes were similarly decreased (data not shown).

With autoimmune exacerbations in patients with MS, peripheral blood monocytes receive inflammatory cytokine signals and cross the blood-brain barrier, with T cells leading to CNS lesions (50, 51). While macrophages are the dominant cell type in these lesions, blood monocytes also extravasate into the CSF and differentiate into CSF macrophages (52). However, it is critical to avoid hyperactivating CSF macrophages in the process of phagocytosing waste to prevent downstream immune activation of autoreactive lymphocytes (53). With B cell depletion, the decrease in myeloid inflammatory cytokines enables CSF macrophages to receive proresolving signals to execute their homeostatic function in mediating tissue repair (54). Our data indicate an antiinflammatory phenotype in CSF macrophages after B cell depletion treatment and that anti-CD20 depletion therapy restored macrophage gene signatures similar to those of healthy controls. Importantly, the inclusion of healthy donor CSF samples allowed us to conclude that anti-CD20 treatment reprogrammed CSF macrophages toward a homeostatic or healthy state.

In the peripheral immune compartment, we uncovered a ubiquitous TNF-α/NF-κB activation signature across a wide range of circulating immune cells following B cell depletion. CD14^+^CD16^+^ monocytes have been shown to be a potent producer of TNF-α (55), and our study suggests that TNF-α is a pleiotropic cytokine that potentially exerts antiinflammatory effects after B cell depletion. B cell depletion confers moderate clinical effectiveness in treating rheumatoid arthritis, whereas anti–TNF-α clearly worsens MS immunopathogenesis (56). Although the increased production of TNF-α with a clinically effective treatment appears to be counterintuitive, the beneficial role of TNF-α in MS is substantiated by clinical trials showing that anti–TNF-α treatment in patients leads to significant worsening of disease activity (57). Moreover, molecular dissection of the MS risk allele rs1800693 located in the gene encoding TNFR1 reveals the associated variant codes for a soluble form of TNFR1, which mimics TNF-α–blocking molecules (58, 59), again consistent with the observation that TNF-α blockade leads to increased disease activity. In the context of chronic inflammation, this scenario in which TNF-α bears an antiinflammatory role is reminiscent of the antitumor immune response. Specifically, in the tumor microenvironment, constant exposure to TNF-α leads to immunosuppressive responses involving Tregs, B regulatory cells, and myeloid-derived suppressor cells (60), and blocking TNF-α leads to an improved response to immune checkpoint blockade in an orthotopic melanoma mouse model (61). Additionally, identifying the precise signaling events leading to the observed transcriptomic changes is challenging, given that the main TNF-α signaling pathway is through NF-κB, a highly ubiquitous signaling pathway. Thus, we cannot exclude the possibility that other receptors signaling through NF-κB were participating to the transcriptomic alterations observed after treatment. Nevertheless, our rich dataset provides a nonbiased roadmap for further mechanistic investigation in animal models.

Several of the established cell annotation methods that we applied were unable to detect changes in the CD4^+^ T cell compartment. The biologically relevant signals could potentially be obfuscated because of the small frequency of myelin-specific T cells. By applying reference mapping with finer granular reference and gene program quantification by NMF, we were able to detect B cell depletion–mediated modulation of various T cell subsets. Although we did not focus on CD8^+^ T cells, we and others previously showed that B cell depletion leads to a decrease in memory CD8^+^CD20^+^ and central memory CD8^+^ T cells (62, 63). Thus, we examined CD20 expression on myelin tetramer–binding T cells. However, even in pre–B cell depletion samples, there were no detectable CD20^+^ cells in the tetramer^+^ T cell population. Additionally, the loss of memory CD8^+^ T cells correlated with lower expression of CXCR3 CNS-related Lymphocyte function-associated antigen-1 integrin, as well as a reduced antiviral cellular immune response (64). On the other hand, the frequency of CD4^+^ Tem cells decreased after ocrelizumab treatment, which is consistent with previous results showing a decrease in CD4^+^ Tem cells (65). In particular, we demonstrate the ability of ocrelizumab to regulate activated autoreactive T cells by showing a significant decrease in the frequency of CD4^+^CCR6^+^ and CD4^+^CXCR5^+^ T cells among cells binding to the myelin tetramer. While we were able to confirm the increase of Treg Eff frequency by both transcriptomic and protein expression, it will be of interest to more precisely identify whether there are differences in myelin-specific and other antigens, as recent studies indicate that EBV infection of B cells is associated with the onset of MS (66).

Our T cell data suggest several mechanisms in which B cell depletion can lead to modulation of T cell functionality. One potential mechanism is that TNF-α from myeloid cells engages TNFR2 on Tregs, leading to suppression of autoreactive T cells after B cell depletion in patients with RRMS. It is established that for Treg-mediated immunosuppression, the induction of an appropriate effector phenotype is essential (67, 68). In this study, we revealed that ocrelizumab treatment induced the formation of a Treg effector phenotype at both mRNA and protein levels and increased the number of Tregs. Hence, the enhancement of Treg function following B cell depletion may have contributed to the therapeutic effect. Alternatively, as first shown by Lanzavecchia et al. (69), B cells may be the key APC, and their depletion may result in loss of autoreactive effector or memory T cells. Similarly, the decrease in MHC expression on myeloid cells may also support this hypothesis. In summary, our systems analysis identified a series of immunoregulatory pathways associated with B cell depletion. This is perhaps not surprising, as the genetic architecture of MS and other autoimmune diseases suggests that multiple pathways are involved in disease pathogenesis (70). It is possible that different immunosuppressive pathways become activated in patients, leading to marked decreases in autoreactive, myelin-reactive T cells in the blood compartment. Clinically, it will be relevant to connect those immune parameters to potential changes in long-term disease progression.

Our analyses comparing immune cells in CSF and blood also highlight distinct changes across compartments, suggesting that regulation of CNS homing mechanisms could be affected by anti-CD20 therapies. Considering the potential disease-mediating and homeostatic functions in the myeloid compartment, future analyses can be designed with a myeloid cell focus using fresh tissue for higher sensitivity in protein detection. Nevertheless, these datasets are exploratory and provide a critical starting point that will require well-designed in vitro and ex vivo assays and appropriate animal models to fully elucidate the perturbational effects of B cell depletion on the functionality of the immune system.

## Methods

### Sex as a biological variable

Our study examined men and women participants, and similar findings are reported for both sexes.

### Patient cohorts

All patients had early-onset RRMS and had not been on previous immunomodulatory treatments. A small subset of patients had received i.v. solumedrol within 3 months of the blood draw. Eighteen patients undergoing scRNA-Seq studies had CSF analysis prior to the initiation of treatment, and 5 of those individuals underwent repeat lumbar punctures, as outlined in Results. A total of 6 age-matched healthy controls underwent lumbar punctures, and those results were previously reported (50). Patient pre-treatment CSF samples were obtained for clinical diagnosis, and patient post-treatment and healthy donor CSF samples were obtained under voluntary enrollment into our research study. Samples were collected at disease onset and prior to scheduled B cell depletion infusions (on the same day), with the exception of 3 patients: samples from patients MS1189, MS1092, and MS1056 were collected 1, 28, and 59 days after infusion, respectively. Samples from an additional 4 patients were subjected to flow cytometric analysis only. Patient characteristics are summarized in Supplemental Table 1.

### Sample preparation for scRNA-Seq

Fresh patient CSF samples were centrifuged, and cells were immediately processed using 10X Genomics 5Pv1 chemistry. Samples were collected prior to infusion of B cell depletion therapy. In the CSF sample cohort, 4 of the patients were administered ocrelizumab B cell depletion treatment, and 1 was treated with rituximab. Patient PBMCs were isolated from whole blood using Lymphoprep (STEMCELL Technologies) density-gradient centrifugation. All patients in the PBMC cohort were administered ocrelizumab for B cell depletion. Cryopreserved, patient-matched pre-treatment and 6-month post-treatment PBMCs were thawed and processed within the same experimental batch using 10X Genomics 5Pv1 chemistry. For PBMCs, TCR libraries were generated along with the gene expression libraries.

### scRNA-Seq QC

PBMC and CSF libraries were sequenced at 20,000 read pairs per cell on an Illumina NovaSeq instrument. Fastq files were processed using Cell Ranger, version 3.1.0, mapping to GRCh38 human reference genome. Alignment and quantitation were performed with the “cell ranger count” command for each emulsion (using the 2020-A 10X Genomics human reference) to generate unique molecular identifier (UMI) count matrices.

For CSF, Data QC was performed in R using the Seurat package. Low-quality cells were filtered out on the basis of mitochondria percentages, UMI counts, and the number of features for individual samples. Samples were then merged, log_10_ transformed, and batch corrected using Harmony.

For PBMCs, extreme outliers were first filtered out by excluding droplets with fewer than 1,500 UMI counts or fewer than 850 unique genes detected. As distribution of those parameters varied across emulsions, we median centered the log_10_-transformed number of unique genes detected and removed low-quality droplets with fewer than 1,100 unique genes detected or more than 2.5% mapping of UMI counts mapping to mitochondrial genes. We also removed potential doublets by filtering out droplets with more than 2,600 unique genes detected.

### scRNA-Seq analysis

#### Dimensionality reduction and clustering.

For cells passing quality control (QC), UMI counts were normalized by dividing each count by the total number of counts per cell. The normalized counts were then multiplied by 10,000, and a pseudocount of 1 was added before log transformation. The stabilized variance of each gene was computed using the variance-stabilizing transformation (VST), and genes with a stabilized variance of greater than 1 were retained for principal component analysis (PCA). Genes mapping to the T cell receptor (TCR), the B cell receptor (BCR), and the Y chromosome were excluded from the PCA analysis. The first 50 principal components (PCs) were computed using a partial singular value decomposition method based on the implicitly restarted Lanczos bi-diagonalization algorithm (IRLBA), as implemented in the Seurat R package (71). To correct for systematic differences across samples, harmony integration (72) was applied to the first 50 PC loadings, and 30 harmony-corrected PCs were retained to build nearest-neighbor graphs for visualization using uniform manifold approximation and projection (UMAP) (minimum distance = 0.5, spread = 10), and community detection using the Louvain algorithm, as implemented in Seurat. A relative likelihood of cells observed in specific experimental conditions was also computed using MELD (20). The effects of sample-level variables (i.e., before vs. after treatment) were quantified across the transcriptomic space using harmony-corrected PC loadings as input. MELD utilizes graph signal processing to model the cellular state space as a network, connecting cells with similar transcriptomic profiles. It generates an enhanced experimental signal (EES) to estimate the likelihood of observing cells from each condition at every point in the manifold. This continuous measure facilitates the derivation of cell subsets that are affected by the sample-level conditions.

Cells were embedded into 2 UMAP dimensions, and the Louvain algorithm was applied. Cluster cell types were annotated on the basis of individual gene expression and the SingleR automatic annotation package using the MonacoImmuneData PBMC reference (73). Variation in cluster frequencies was tested in the CSF and in the blood separately by modeling the per-sample cluster frequencies using a β distribution in a generalized linear model framework, as implemented in the betareg R package, using the logit link function (74). We also used an alternative constrained β binomial model based on counts, implemented in the sccomp R package (75), which yielded similar results (see Supplemental Data 1 and Supplemental Data 3).

#### DEG analysis.

For DEG testing at the single-cell level in CSF, we used a fixed-effect negative binomial model, as implemented in the DESeq2 package (76), supplemented with a recent optimization for scRNA-Seq data (77). Genes with low expression levels were excluded on the basis of a UMI count per cell of less than 0.005, as well as ribosomal, mitochondrial, TCR, and BCR counts. Differences in counts were evaluated with a treatment or disease status predictor, with the addition of categorical donor information as covariates. Shrunken log fold changes were then computed using adaptive shrinkage methods implemented in the ashr R package (78) (using a mixture of normal distributions). *P* values were computed by fitting a reduced model and using a likelihood ratio test, and multiple-comparison correction was performed by FDR (Benjamini-Hochberg [BH] method) across all genes tested, as implemented in the DESeq2 package. Genes with a FDR below 0.1 were considered differentially expressed. For differential expression testing at the single-cell level in PBMCs, a negative binomial mixed linear model was used, as implemented in the NEBULA package (31). Shrunken log fold changes were then used as ranking metrics to run GSEA.

#### Treg volcano and ligand-receptor analyses.

Volcano plots displaying differential expression analysis were created using NEBULA, comparing pre- and post-treatment Treg populations. Among genes with differential expression (BH <0.05) and an average expression level of greater than 0.1, the genes that encode surface proteins (based on the Cell Surface Protein Atlas [CSPA] surfaceome protein database) were selectively labeled (79). NicheNet (nichenetr) (80) was used to identify predicted ligand-receptor interactions between myeloid cell populations and Tregs, with a particular focus on potential ligands that are differentially regulated in myeloid cells with B cell depletion treatment. Tregs were selected as the “receiver cell type,” including all expressed genes as potential receptors. Myeloid cells were selected as the “sender cell type,” limiting the set of potential ligands to the combined list of genes differentially expressed with B cell depletion treatment in myeloid cell clusters (see differential expression analysis). Predicted ligand-receptor interactions are displayed as a heatmap in which ligands (expressed by myeloid cells) were plotted against receptors (expressed by Tregs) and weighted by prior interaction potential.

#### CD4^+^ T cell automatic labeling and quantification of gene programs.

PBMC and CSF data were processed using the pipeline developed in a previous study (37) to assign CD4^+^ T cell clusters. This pipeline uses Azimuth (44) for the extraction of CD4^+^ T cells and uses Symphony (81) for prediction of CD4^+^ T cell clusters. For interpretability, “Treg Act” was renamed to “Treg Int” from the original literature. We tested for variation in cluster frequency by modeling the per-sample cluster frequencies using a β distribution in a generalized linear model framework, as implemented in the betareg R package. For the assessment of TIGIT protein expression, we used cellular indexing of transcriptomes and epitopes by sequencing (CITE-Seq) data from PBMCs deposited in GSE164378 and performed reference mapping using the pipeline. Additionally, a 12-dimensional qualitative evaluation was conducted on the extracted CD4^+^ T cells using NMFproj (82). A generalized linear model was applied to assess feature changes per cluster (37).

### Flow cytometric analysis

Frozen PBMCs were used for flow cytometry validation, except longitudinal myelin tetramer stainings were performed on fresh PBMCs (*n* = 4). Patient PBMCs were stained with ViaKrome 808 Fixable Viability dye (Beckman Coulter) following the manufacturer’s instructions. Cells were then labeled with surface antibodies for 30 minutes at 4°C. For intracellular staining, cells were fixed and permeabilized with BD Cytofix/Cytoperm Buffer (BD Biosciences) for 10 minutes at room temperature and then washed with PBS. Intracellular proteins were stained in permeabilization buffer (eBioscience) for 30 minutes at 4°C. Antibody details are provided in Supplemental Table 2. For TNF-α staining, monocytes were enriched from cryopreserved PBMCs using the EasySep Human Monocyte Enrichment Kit without the CD16 Depletion Kit (STEMCELL Technologies). Enriched monocytes were stimulated with 100 ng/mL LPS for 4 hours at 37°C before staining. To investigate myelin tetramer–reactive T cells, APC- or PE-conjugated tetramers, which were composed by DRB1*15:01 (loaded with MBP, MOG, and PLP) or DRB1*04:01 (loaded with MOG and PLP), were used (4, 48). Myelin tetramers were incubated with cells for 30 minutes at 37°C before staining with antibodies. Cells were acquired on a BD Symphony flow cytometer with FACSDiva (BD Pharmingen), and data were analyzed with FlowJo software, version 10 (Treestar). Changes in frequencies were tested using the betareg model, as described in “scRNA-Seq analysis.” Changes in MFI were tested using a generalized linear model with Gaussian distribution and a log link function.

### Peptide loading

Biotinylated monomers were diluted to a concentration of 0.5 mg/mL with 0.1 M phosphate buffer and incubated with 0.4 mg/mL at 37°C for 72 hours in the presence of 2.5 mg/mL *n*-octyl β-d-glucopyranoside (OG) and 1 mM Pefabloc SC (MilliporeSigma). Peptide-loaded monomers were subsequently conjugated into tetramers using R-Phycoerythrin (R-PE) streptavidin (Thermo Fisher Scientific) or fluorochromes of interest at a molar ratio of 8:1. Myelin peptide sequences are listed in Supplemental Table 3.

### Statistics

All statistical analyses were performed in R programming language using generalized linear models. Where appropriate, we included a donor covariate to model the paired nature of samples (before and after treatment). *P* values were calculated using the Wald test of regression coefficients. A *P* value of less than 0.05 or FDR < 0.1 was considered statistically significant. Where relevant, parametric models effect size were depicted as mean ± SEM. For descriptive distributions representation, boxplot were used with the box representing the 25th, 50th, and 75th percentile and whiskers representing ± 1.5 interquartile range.

### Study approval

This study was approved by the Institutional Review Board at Yale University. CSF and blood samples were obtained with informed consent from healthy donors and patients with MS.

### Data and code availability

All raw scRNA-Seq data generated in this study are deposited in Database of Genotypes and Phenotypes (dbGaP) (phs003938.v1.p1). These newly generated data were analyzed jointly with published scRNA-Seq data on CSF samples from 6 healthy controls and 4 patients with MS at diagnosis (MS1102, MS1131, MS1171, and MS1228) already deposited in dbGAP (phs002222.v1.p1). All code used for genomics analysis is available on github (https://github.com/ImmuneAxisa/Ocrevus_manuscript, commit ID: 80ab0da) and figshare (https://doi.org/10.6084/m9.figshare.28204532), along with intermediate analysis files. Supporting data are available in the Supporting Data Values file.

## Author contributions

JW, JM, IY, TSS, PPA and DAH designed experiments. JM, LZ, KR, NBP, IY, and JP conducted experiments. JW, JM, YY, MED, NS, and EEL collected and analyzed data. CWC, AH, RP, and CR advised on data analyses. TSS, PPA, and DAH supervised data analyses. JW, JM, TSS, PPA, and DAH wrote the manuscript, integrating feedback from all co-authors.

## Supplementary Material

Supplemental data

Supplemental data set 1

Supplemental data set 2

Supplemental data set 3

Supplemental data set 4

Supplemental data set 5

Supplemental data set 6

Supplemental table 1

Supporting data values

## Version 1 03/11/2025

In-Press Preview

## Version 2 06/02/2025

Electronic publication
