# Supplementary material for: Transcriptomic profiling after B cell depletion reveals central and peripheral immune cell changes in multiple sclerosis
Source: J Clin Invest. 2025 Mar 11;135(11):e182790. doi: 10.1172/JCI182790 (PMC12126227; doi:10.1172/JCI182790)
Supplement: Supplemental data [file jci-135-182790-s085.pdf]

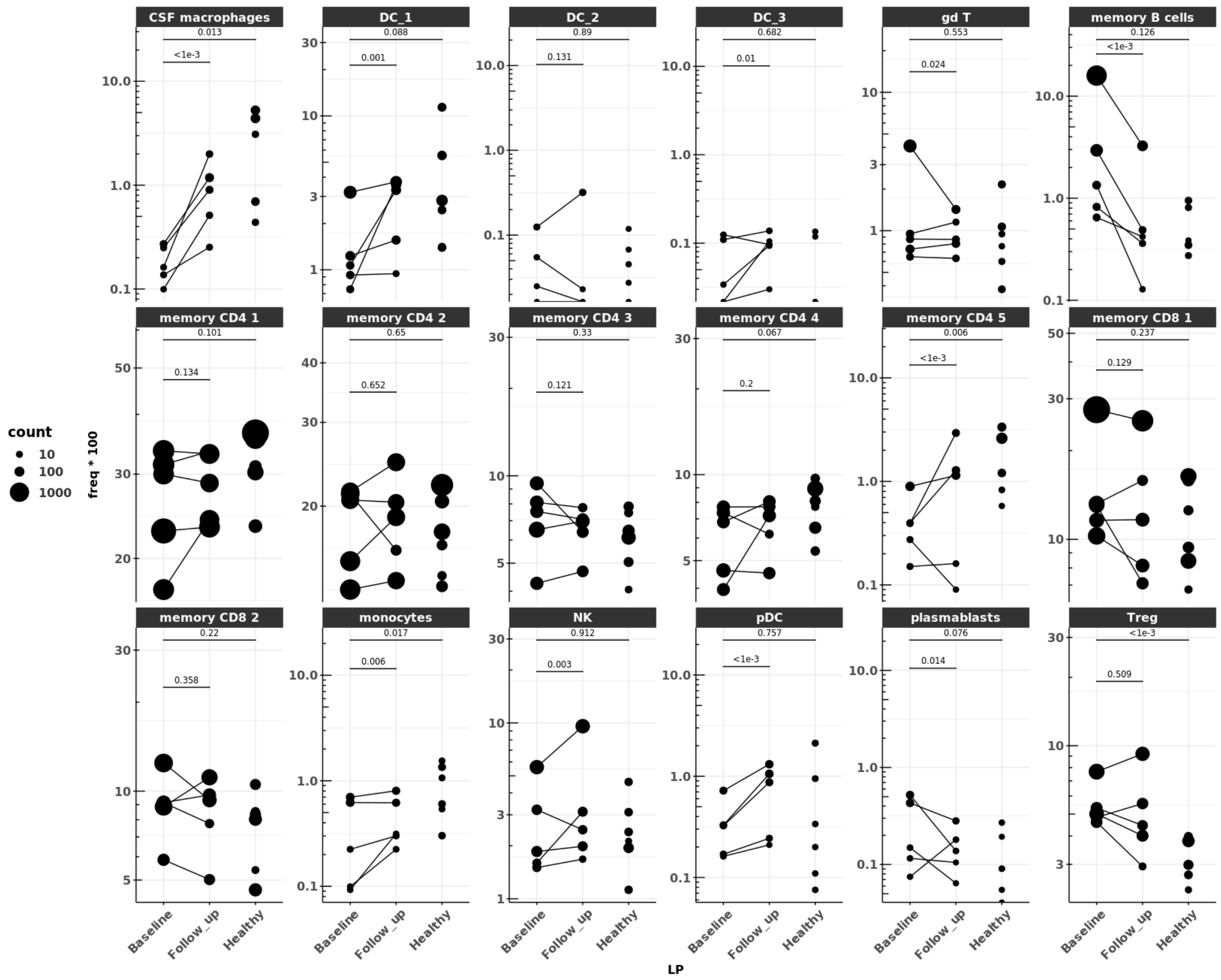

Supplemental Figure 1. Cluster frequency changes in the CSF.

Per sample cluster frequencies in baseline MS patients with paired follow up samples (n=5) and healthy donors (n=6)

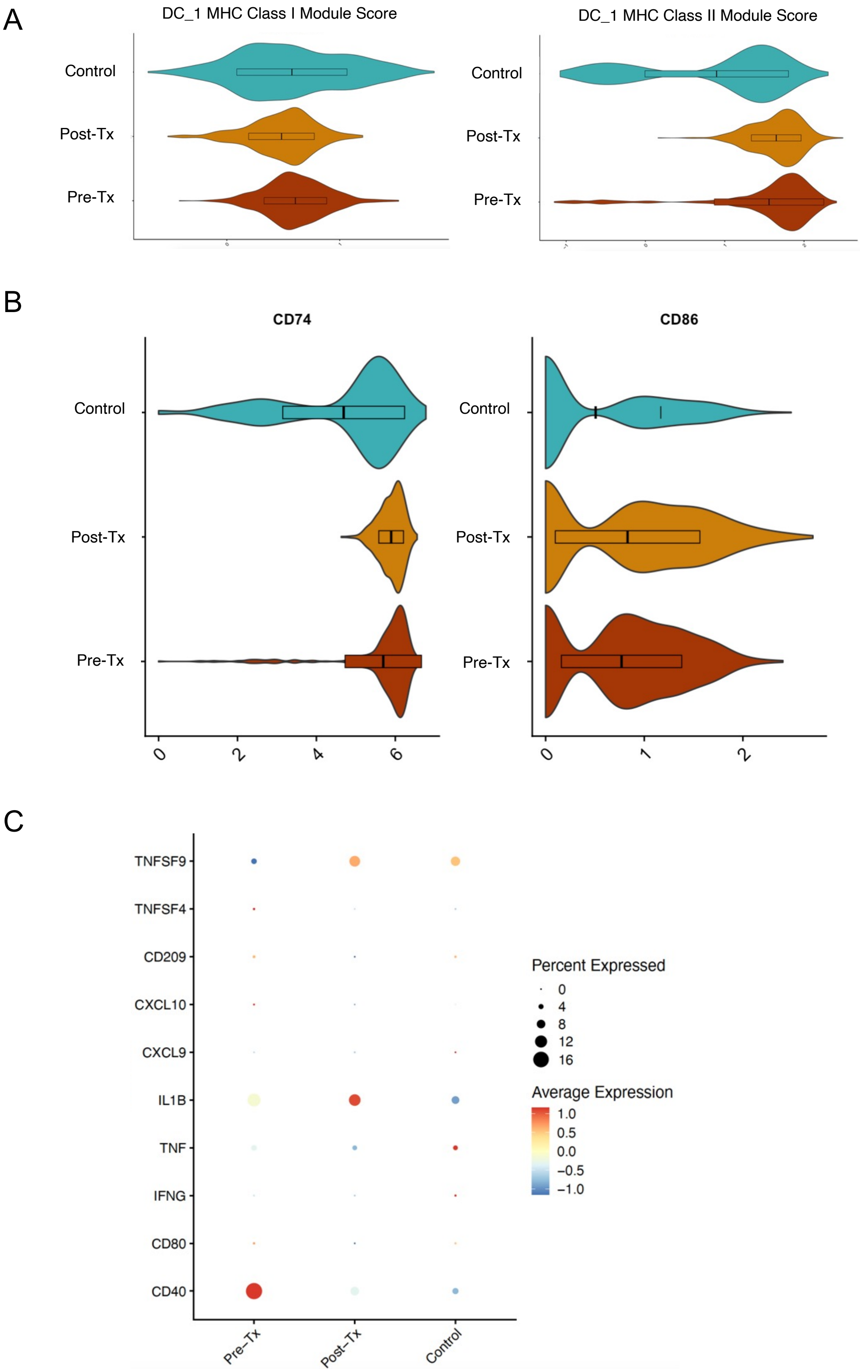

A
DC_1 MHC Class I Module Score
DC_1 MHC Class II Module Score
Control
Post-Tx
Pre-Tx
B
CD74
CD86
Control
Post-Tx
Pre-Tx
0
2
4
6
1
C
TNFSF9
TNFSF4
CD209
CXCL10
CXCL9
IL1B
TNF
IFNG
CD80
CD40
Pre–Tx
Post–Tx
Control
Percent Expressed
0
4
8
12
16
Average Expression
1.0
0.5
0.0
–0.5
–1.0

Supplemental Figure 2. Transcriptomic characterization of CSF DC_1 cluster.
(A) MHC class I and class II gene module scores in healthy, pre- and post- treatment CSF samples. **(B)** CD74 and CD86 gene expression in healthy, pre- and post- treatment CSF samples. **(C)** Expression level and percent of expression of inflammatory dendritic cell genes.

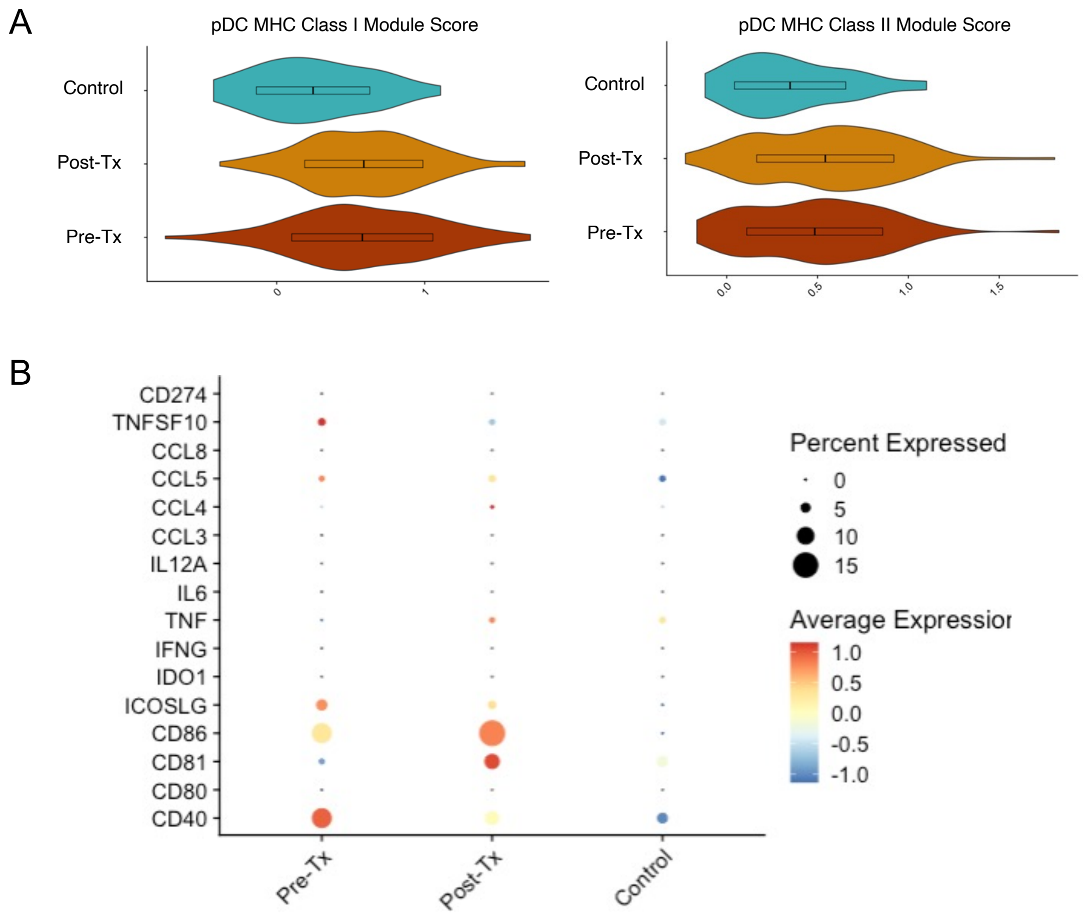

Supplemental Figure 3. Transcriptomic characterization of CSF pDC cluster.
(A) MHC class I and class II gene module scores in healthy, pre- and post- treatment CSF samples. **(B)** Individual gene expression in healthy, pre- and post- treatment CSF samples.

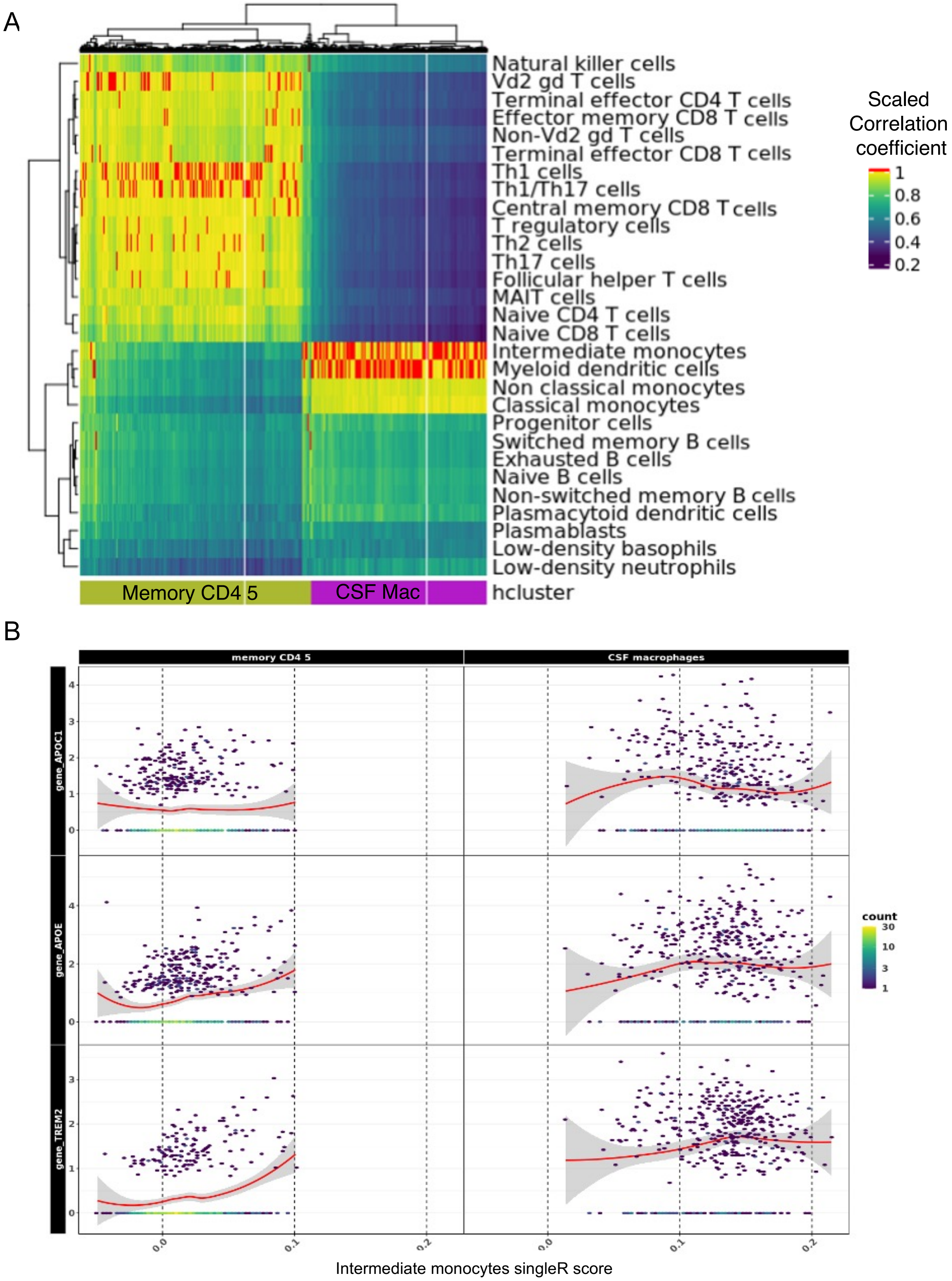
A
Natural killer cells
Vd2 gd T cells
Terminal effector CD4 T cells
Effector memory CD8 T cells
Non-Vd2 gd T cells
Terminal effector CD8 T cells
Th1 cells
Th1/Th17 cells
Central memory CD8 T cells
T regulatory cells
Th2 cells
Th17 cells
Follicular helper T cells
MAIT cells
Naive CD4 T cells
Naive CD8 T cells
Intermediate monocytes
Myeloid dendritic cells
Non classical monocytes
Classical monocytes
Progenitor cells
Switched memory B cells
Exhausted B cells
Naive B cells
Non-switched memory B cells
Plasmacytoid dendritic cells
Plasmablasts
Low-density basophils
Low-density neutrophils
Memory CD4 5
CSF Mac
hcluster
Scaled Correlation coefficient
1
0.8
0.6
0.4
0.2
B
memory CD4 5
CSF macrophages
gene_APOC1
gene_APOE
gene_TREM2
count
30
10
3
1
Intermediate monocytes singleR score

Supplemental Figure 4. Common CSF signature between macrophage and T cell clusters. **(A)** Community based clustering (Louvain) on shared nearest neighbor graph led to a cluster with mixed lineages (T cells and myeloid cells), based on singleR scoring against the Monaco reference. Separation of the two lineages by hierarchical clustering let us define two clusters for downstream analysis. **(B)** ‘Intermediate monocytes’ singleR score separates each lineage, but both lineages express similar gene signature associated with CSF residency (*APOC1, APOE, TREM2*).

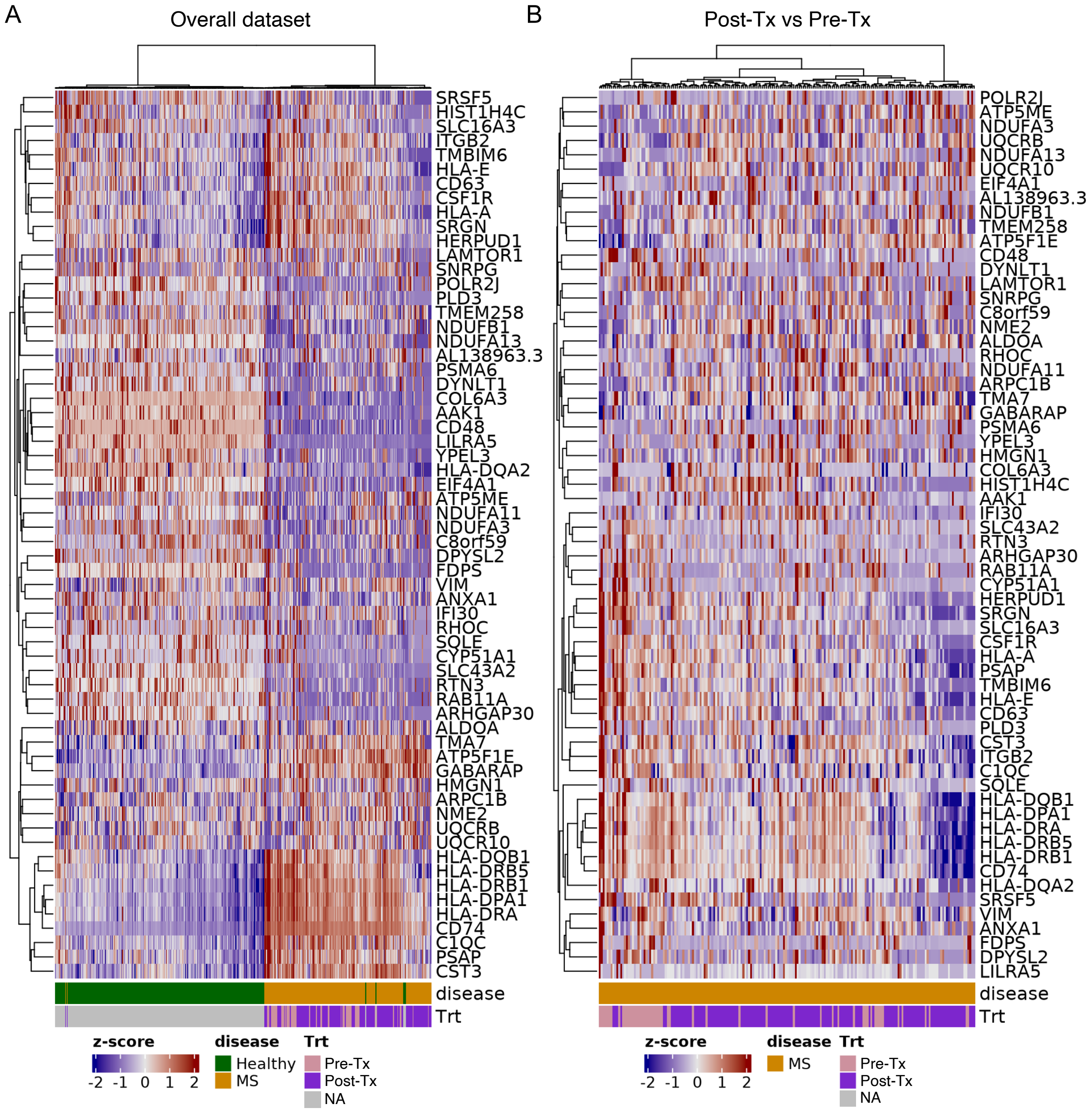

Supplemental Figure 5. Transcriptomic changes in CSF macrophages.
(A and B) Heatmap of differentially expressed genes in the 'Mac 1' CSF macrophage cluster for MS baseline vs healthy donor (A) and MS follow up vs baseline (B).

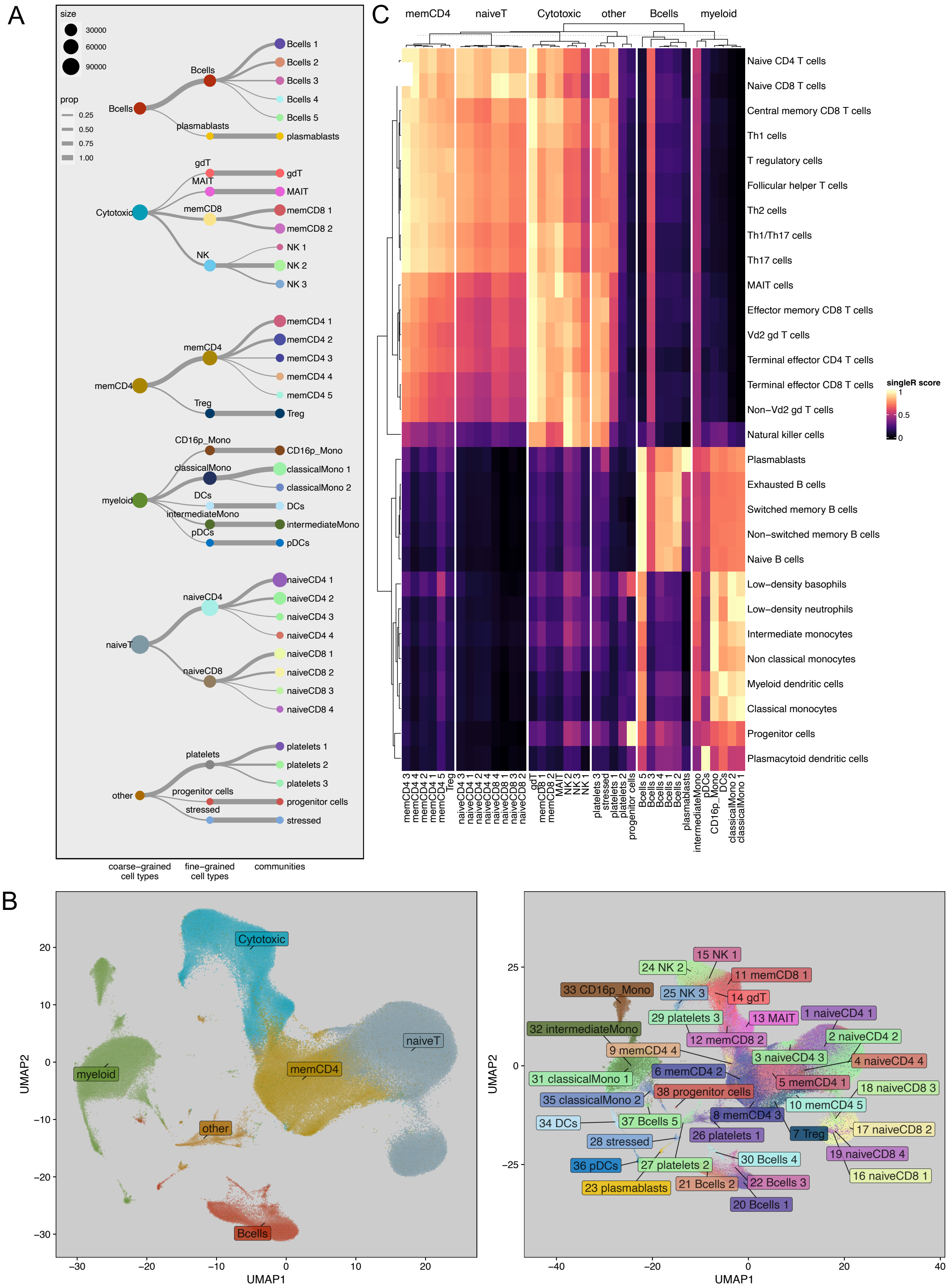
A
size
30000
60000
90000
prop
0.25
0.50
0.75
1.00
Bcells
Bcells 1
Bcells 2
Bcells 3
Bcells 4
Bcells 5
plasmablasts
Cytotoxic
gdT
MAIT
memCD8
memCD8 1
memCD8 2
NK
NK 1
NK 2
NK 3
memCD4
memCD4 1
memCD4 2
memCD4 3
memCD4 4
memCD4 5
Treg
myeloid
CD16p_Mono
classicalMono
classicalMono 1
classicalMono 2
DCs
intermediateMono
pDCs
naiveT
naiveCD4
naiveCD4 1
naiveCD4 2
naiveCD4 3
naiveCD4 4
naiveCD8
naiveCD8 1
naiveCD8 2
naiveCD8 3
naiveCD8 4
other
platelets
platelets 1
platelets 2
platelets 3
progenitor cells
stressed
coarse−grained cell types
fine−grained cell types
communities
C
memCD4
naiveT
Cytotoxic
other
Bcells
myeloid
Naive CD4 T cells
Naive CD8 T cells
Central memory CD8 T cells
Th1 cells
T regulatory cells
Follicular helper T cells
Th2 cells
Th1/Th17 cells
Th17 cells
MAIT cells
Effector memory CD8 T cells
Vd2 gd T cells
Terminal effector CD4 T cells
Terminal effector CD8 T cells
Non−Vd2 gd T cells
Natural killer cells
Plasmablasts
Exhausted B cells
Switched memory B cells
Non−switched memory B cells
Naive B cells
Low−density basophils
Low−density neutrophils
Intermediate monocytes
Non classical monocytes
Myeloid dendritic cells
Classical monocytes
Progenitor cells
Plasmacytoid dendritic cells
singleR score
1
0.5
0
B
Cytotoxic
naiveT
myeloid
memCD4
other
Bcells
UMAP1
UMAP2
15 NK 1
24 NK 2
11 memCD8 1
33 CD16p_Mono
25 NK 3
14 gdT
29 platelets 3
13 MAIT
1 naiveCD4 1
32 intermediateMono
12 memCD8 2
2 naiveCD4 2
9 memCD4 4
3 naiveCD4 3
4 naiveCD4 4
31 classicalMono 1
6 memCD4 2
5 memCD4 1
35 classicalMono 2
38 progenitor cells
18 naiveCD8 3
10 memCD4 5
34 DCs
37 Bcells 5
8 memCD4 3
28 stressed
26 platelets 1
7 Treg
17 naiveCD8 2
30 Bcells 4
19 naiveCD8 4
36 pDCs
27 platelets 2
16 naiveCD8 1
23 plasmablasts
21 Bcells 2
22 Bcells 3
20 Bcells 1

Supplemental Figure 6. Community detection and aggregation of PBMC scRNAseq dataset.

(A) initial community detection resolution (coarse-grained cell type) and subsequent nested community detection (community). Communities were then merged into fine-grained cell types. **(B)** UMAP embedding annotated with coarse-grained cell types assignments and communities assignments. **(C)** singleR scores heatmap for fined-grained cell types, grouped by coarse-grained cell types.

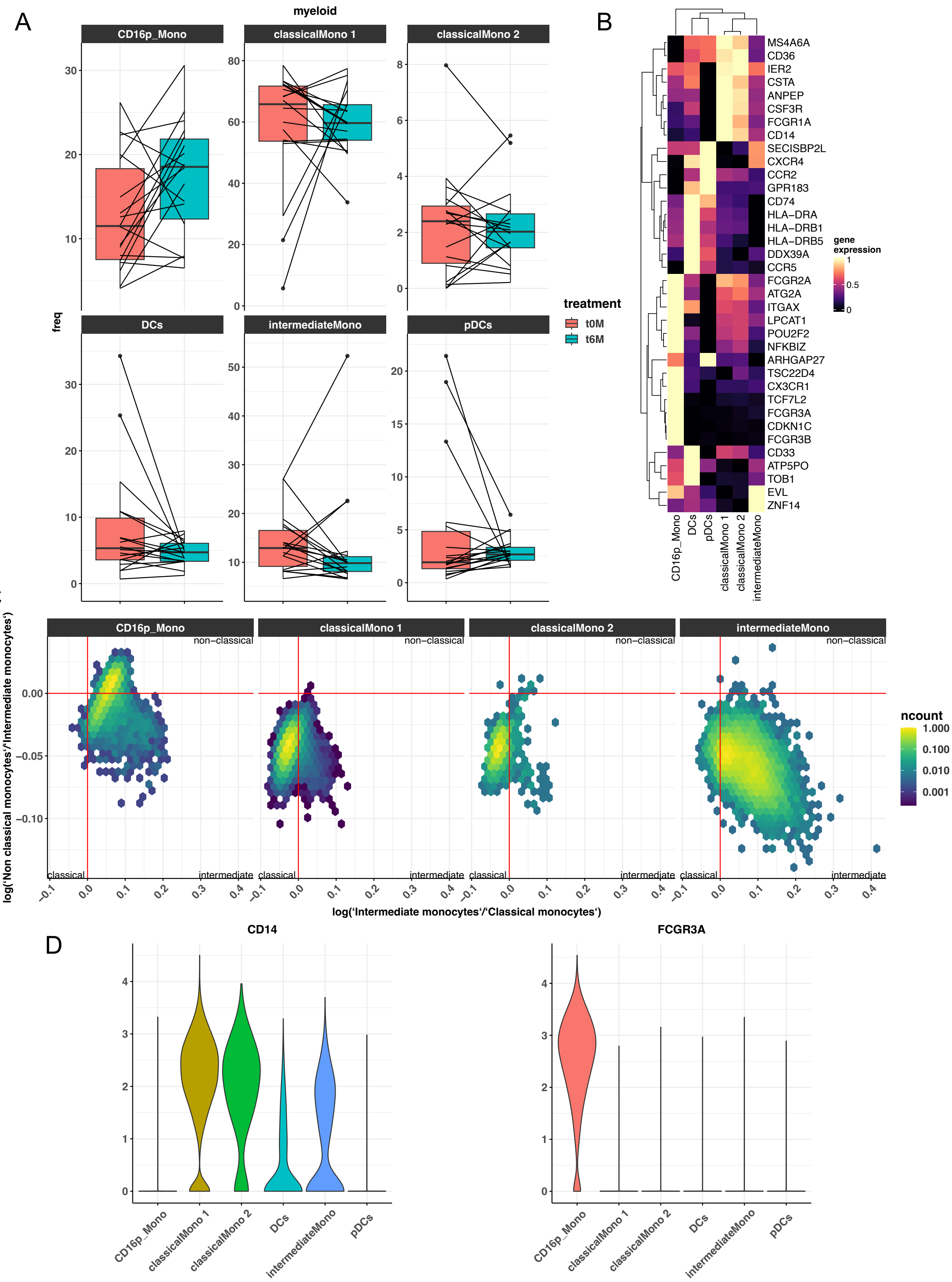
A
myeloid
CD16p_Mono
classicalMono 1
classicalMono 2
DCs
intermediateMono
pDCs
freq
treatment
t0M
t6M
B
MS4A6A
CD36
IER2
CSTA
ANPEP
CSF3R
FCGR1A
CD14
SECISBP2L
CXCR4
CCR2
GPR183
CD74
HLA−DRA
HLA−DRB1
HLA−DRB5
DDX39A
CCR5
FCGR2A
ATG2A
ITGAX
LPCAT1
POU2F2
NFKBIZ
ARHGAP27
TSC22D4
CX3CR1
TCF7L2
FCGR3A
CDKN1C
FCGR3B
CD33
ATP5PO
TOB1
EVL
ZNF14
gene expression
CD16p_Mono
DCs
pDCs
classicalMono 1
classicalMono 2
intermediateMono
C
CD16p_Mono
classicalMono 1
classicalMono 2
intermediateMono
non−classical
classical
intermediate
log('Non classical monocytes'/'Intermediate monocytes')
log('Intermediate monocytes'/'Classical monocytes')
ncount
1.000
0.100
0.010
0.001
D
CD14
FCGR3A
CD16p_Mono
classicalMono 1
classicalMono 2
DCs
intermediateMono
pDCs

Supplemental Figure 7. Characterization of myeloid clusters in PBMC dataset.
(A) frequency changes in myeloid clusters in MS follow up vs baseline samples. **(B)** heatmap of myeloid cell type gene markers across fine-grained cell types. **(C)** singleR monocytes subtypes score comparison across clusters defines 3 quadrants to differentiate between classical, intermediate, and non-classical subsets. CD16+ monocytes cluster presents with a mixture of intermediate and non-classical cells. **(D)** RNA expression of CD14 and FCGR3A (encoding CD16) in myeloid clusters.

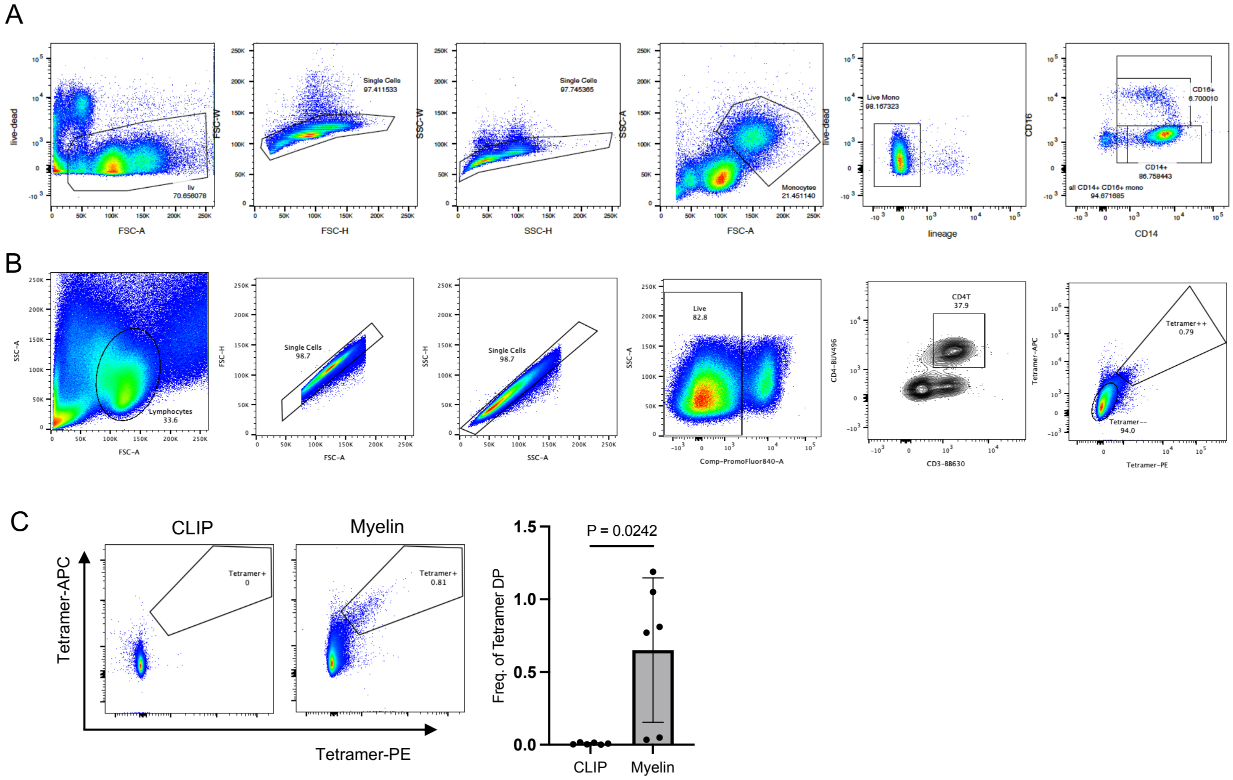

Supplemental Figure 8. Flow cytometry gating strategy and establishment of control. **(A and B)** Flow cytometry images showed the gating strategy for monocytes (A) and lymphocytes (B). **(C)** As a negative control, an MHC tetramer loaded with peptide of Class II-associated invariant chain peptide (CLIP) was used (n=6).

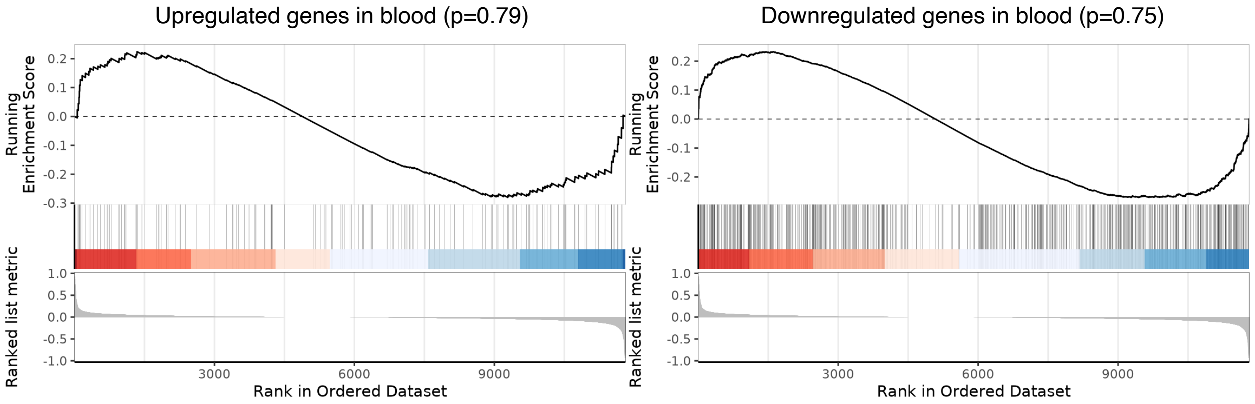

Supplemental Figure 9. GSEA between blood monocytes and CSF macrophages.
GSEA on differential gene expression output from CSF macrophage cluster using custom genesets composed of upregulated and downregulated genes in PBMC cluster 33 of CD16+ monocytes. The absence of significant enrichment suggests distinct dynamics between the two compartments.

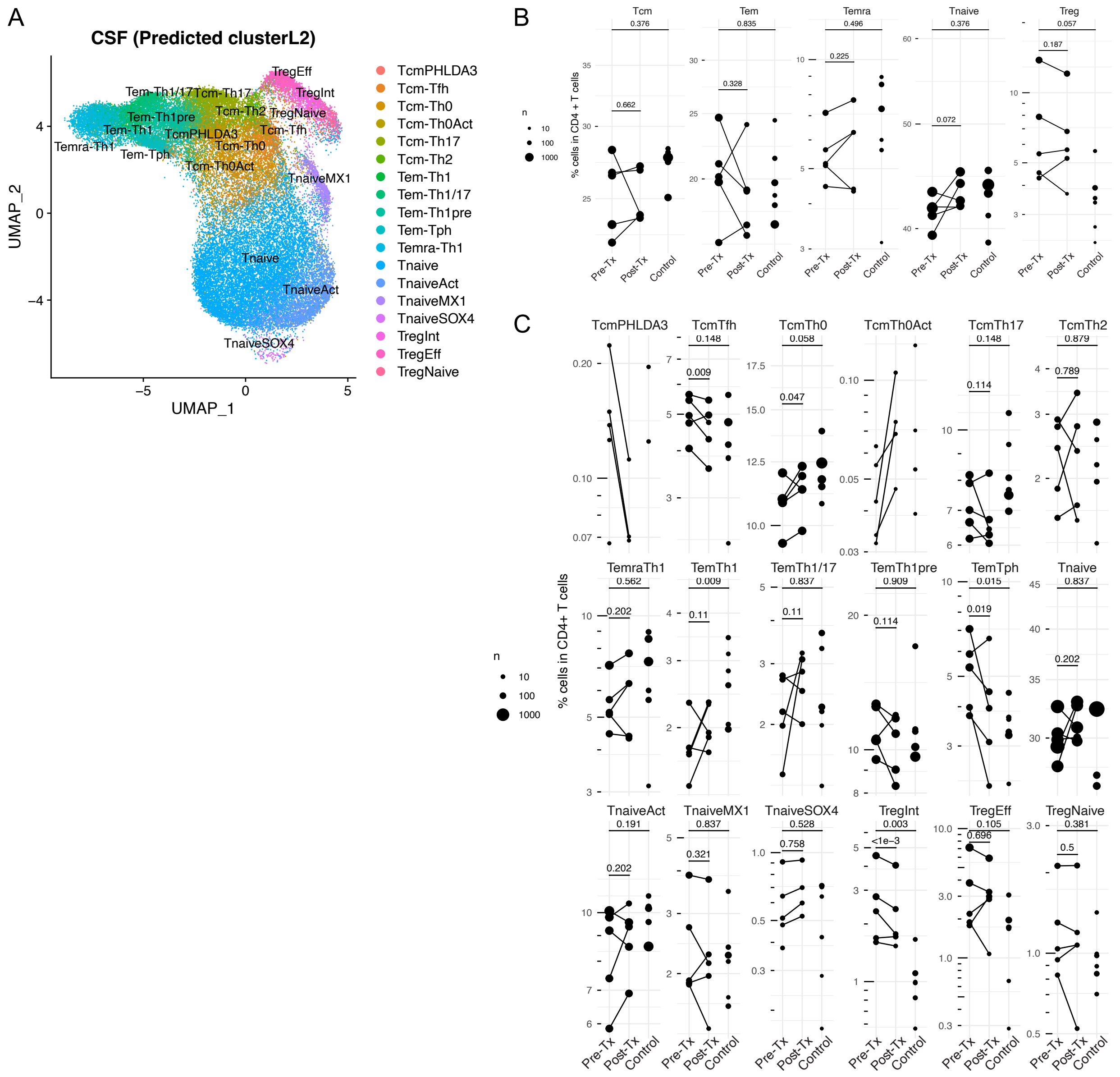

Supplemental Figure 10. CD4+ T cell alterations in CSF

(A) Inferred CSF CD4$^+$ T cell clusters on UMAP plot. The clusters were assigned to a detailed cluster (cluster L2) level. **(B and C)** CSF CD4$^+$ T cell frequency changes after anti-CD20 treatment in cluster L1 level (B) and cluster L2 level (C). Coefficients of cell frequency change per cluster L2 quantified GLM (method) are visualized on the UMAP plot (left). CD4$^+$ T cluster frequency pre- and post-B cell depletion therapy (right).

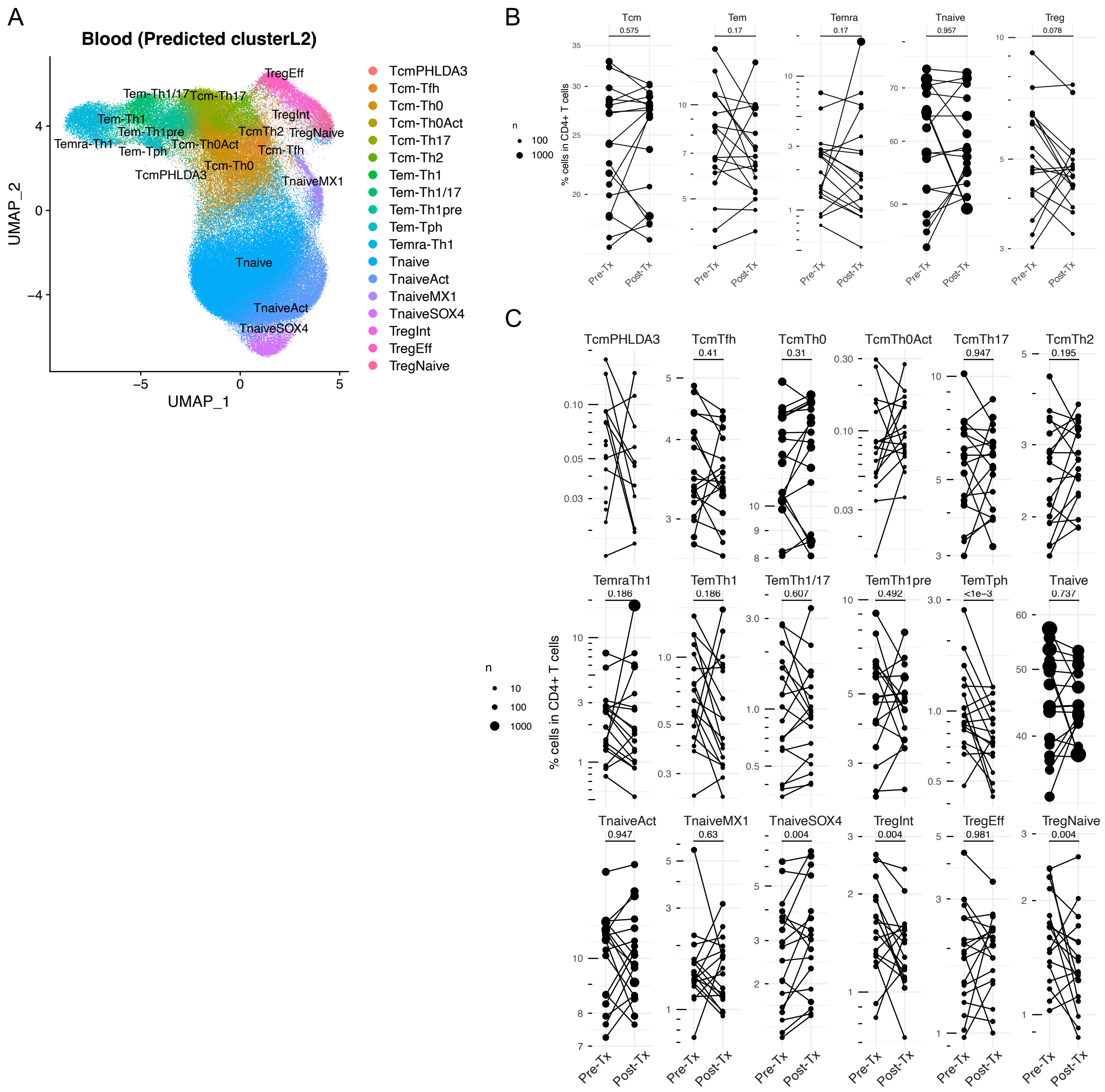

Supplemental Figure 11. CD4+ T cell alterations in blood

(A) Inferred blood CD4^{+} T cell clusters on UMAP plot. The clusters were assigned to a detailed cluster (cluster L2) level. **(B and C)** Blood CD4^{+} T cell frequency changes after anti-CD20 treatment in cluster L1 level (B) and cluster L2 level (C). Coefficients of cell frequency change per cluster L2 quantified GLM (method) are visualized on the UMAP plot (left). CD4^{+} T cluster frequency pre- and post-B cell depletion therapy (right).

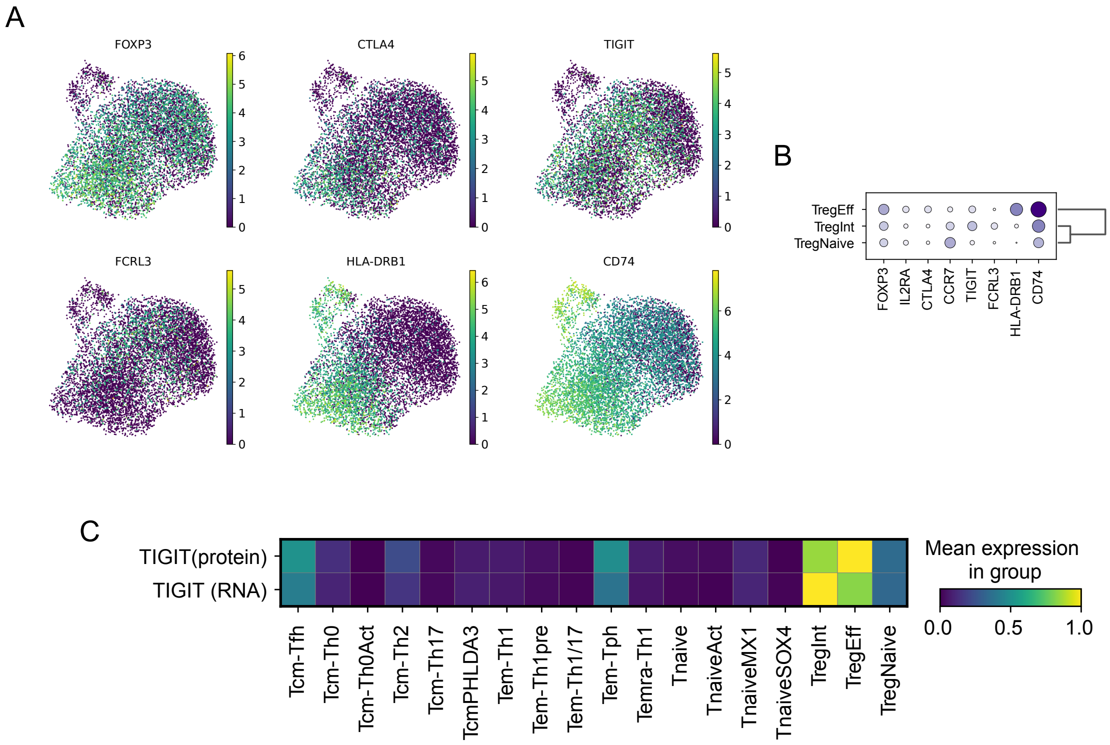

Supplemental Figure 12. Detailed Treg characteristics

(A and B) Treg marker gene expression. Blood Treg cells were extracted and shown using UMAP (A) or dot plot (B). **(C)** Heatmap showing protein and RNA expression of TIGIT in cluster L2 clusters. CITE-seq data (GSE164378) was used for the visualization.

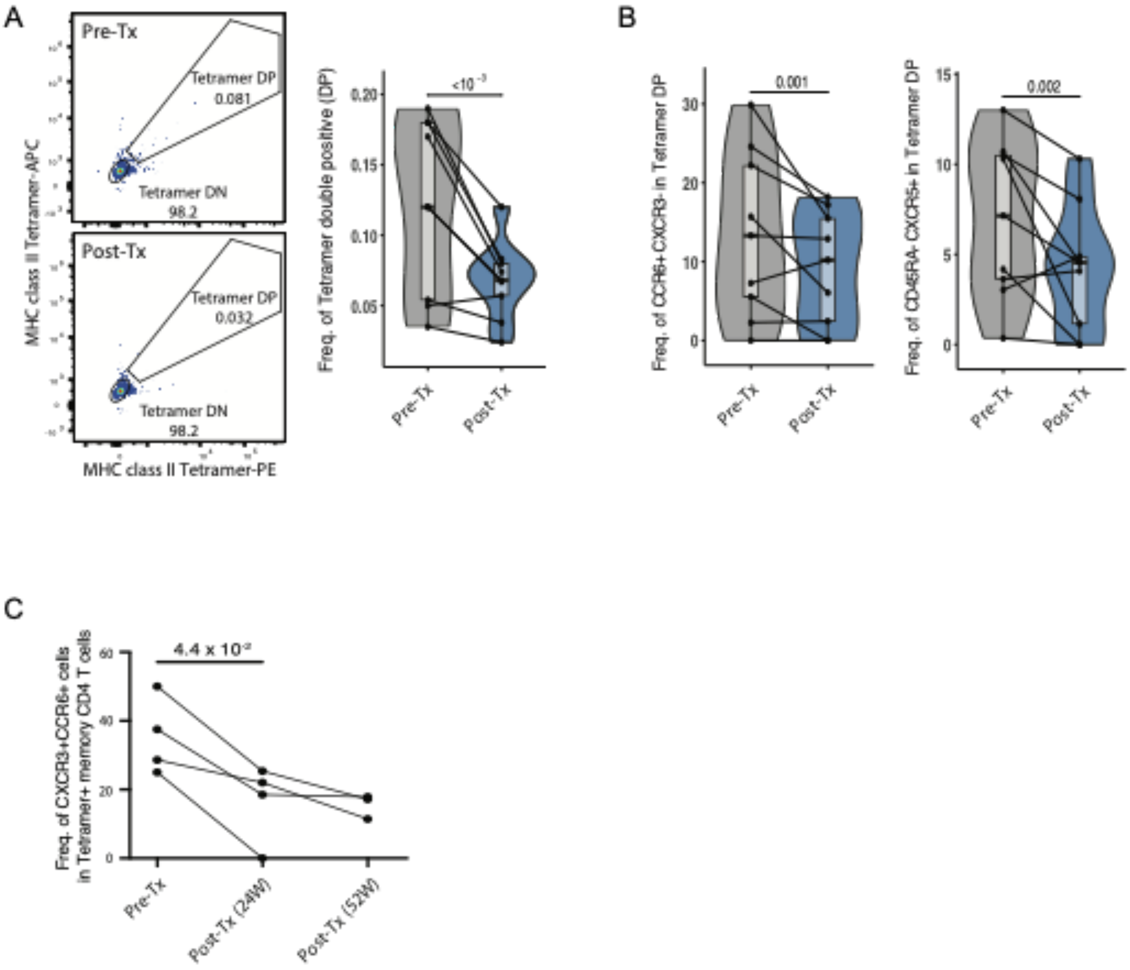

Supplemental Figure 13. Characteristics of myelin tetramer-reactive T cells in different cohorts.

(A) Flow cytometry analysis of myelin tetramer-reactive CD4+ T cell frequency at pre-treatment and 6-month post-treatment timepoints in different MS cohort (n=9). **(B)** Tfh (CD45RA— CXCR5+) cells in tetramer-reactive CD4+ T cells and Th17 (CCR6+CXCR3-) cells in tetramer-reactive CD4+ T cells frequencies at pre-treatment and 6-month post-treatment timepoints (n=9). **(C)** Longitudinal kinetic analysis of the frequency of autoreactive CCR6+CXCR3+ CD4+ T cells using flow cytometry at pre-treatment (n=4), 24-week post-treatment (n=4) and 52-week post-treatment (n=3) timepoints.
